# Identification and functional characterization of a fructose-inducible phosphotransferase system in *Azospirillum brasilense* Sp7

**DOI:** 10.1128/aem.00828-24

**Published:** 2025-01-16

**Authors:** Sushant Rai, Vijay Shankar Singh, Parikshit Gupta, Anil Kumar Tripathi

**Affiliations:** 1 School of Biotechnology, Institute of Science, Banaras Hindu University 30114 , Varanasi, India; 2 Department of Microbiology, School of Life Sciences, Sikkim University 684192 , Gangtok, Sikkim, India; Georgia Institute of Technology, Atlanta, Georgia, USA

**Keywords:** fructose, *Azospirillum brasilense*, phosphotransferase system, type VI secretion system

## Abstract

Plant growth-promoting rhizobacterium *Azospirillum brasilense* Sp7 utilizes fructose efficiently via a fructose phosphotransferase system (Fru-PTS). Its genome encodes two putative Fru-PTS, each consisting of FruB (EIIA), FruK (Pfk), and FruA (EIIBC) proteins. We compared the proteomes of *A. brasilense* Sp7 grown with malate or fructose as sole carbon source, and noticed upregulation of the constituent proteins of Fru-PTS1 only on fructose. Inactivation of *fruA* gene of both the Fru-PTS showed that Fru-PTS1 is the main PTS involved in fructose utilization. Overexpression of *fruA1* in *A. brasilense* Sp7 enhanced its growth on fructose showing improved consumption of fructose. This suggested that fructose utilization in *A. brasilense* Sp7 is limited due to the limitation of EIIBC component. A FruR-type regulator, encoded divergently to the Fru-PTS1 operon, was required for chemotaxis toward fructose. Although not an absolute necessity for the growth of fructose, FruR was required for the optimal growth of fructose. The *fruB1* promoter was activated by fructose and repressed by malate, but FruR does not seem to regulate its expression. A 27-nucleotide stem-loop structure located between the −125 and −99 promoter proximal region of *fruB1* was involved in fructose inducibility and malate repression. Fructose also upregulated several proteins involved in the biogenesis of a Type 6 secretion system. Here, we have shown that *A. brasilense* Sp7 was able to inhibit the growth of *Escherichia coli* and *Agrobacterium tumefaciens* in the presence of fructose, and that an intact T6SS was required for contact-dependent growth inhibition of the two Gram-negative bacteria.

**IMPORTANCE:**

*Azospirillum brasilense*, a plant growth-promoting rhizobacterium, has limited ability to utilize carbohydrates and sugars. Although it is known to utilize fructose via a fructose phosphotransferase system (fructose-PTS), the genes involved in fructose utilization and the role of fructose in its biology were not well characterized. This study has shown that out of the two units of fructose-PTS encoded in its genome, fructose-PTS1 plays the major role in fructose utilization. Overexpression of the membrane component (EIIBC) improved the growth of *A. brasilense* on fructose. The ability of fructose to induce proteins of the Type 6 Secretion System (T6SS) enables *A. brasilense* to cause contact-dependent inhibition of the growth of *Escherichia coli* as well as *A. tumefaciens*. This is the first report on the fructose inducibility of T6SS in *A. brasilense*, which may provide a handle to control the growth of undesirable bacteria using T6SS of *A. brasilense* in a mixed culture.

## INTRODUCTION

Bacteria transport sugars primarily via phosphoenolpyruvate (PEP): carbohydrate phosphotransferase systems (PTS), which phosphorylate sugars when they are transported inside the cell (1). A typical PTS consists of cytoplasmic proteins, enzyme I (EI), a histidine-containing phosphocarrier protein (HPr), and a sugar-specific enzyme II (EII) complex. Both EI and HPr are not sugar-specific and hence used for the transport of most of the PTS sugars. The EII complex consists of two cytosolic domains (EIIA and EIIB) and a transmembrane domain (EIIC). The phosphorylation of sugars begins with autophosphorylation of EI by PEP, followed by the transfer of phosphoryl group from EI to EIIA with the help of HPr. The phosphoryl group is then transferred from EIIA to EIIB, and then to the PTS sugar during its transport into the cell through EIIC (2–4).

Since fructose feeds directly into glycolysis without isomerization or epimerization, it is considered an important sugar in the early evolution of the carbohydrate metabolic pathway in bacteria. It is also the first hexose synthesized through gluconeogenesis (5). It is transported mainly through the fructose-specific PTS (PTS^Fru^) and converted into fructose-1-phosphate (F1P). In bacteria, the occurrence of fructose PTS (PTS^Fru^) is more widespread than any other carbohydrate PTS including the glucose PTS (PTS^Glc^) (6). The PTS^Fru^ of *Escherichia coli* is unique as it possesses its own HPr-like domain, FPr, which is fused to an EIIA domain via a central M domain of unknown function to constitute FruB, which catalyzes phosphotransfer from EI to FruA (EIIB′BC^Fru^), which finally phosphorylates fructose to fructose 1-phosphate (F1P) during its transport inside the bacterial cell. The 1-phosphofructokinase (FruK) then converts F1P to fructose 1,6-bisphosphate (FBP) (7).

Bacteria of the genus *Azospirillum* colonize plant roots of a variety of plants and promote their growth by producing phytohormones and siderophores (8, 9). Several strains of *Azospirillum brasilense* and *A. lipoferum* have been used as crop inoculants for enhancing the yield of several non-legumes crops such as corn, rice, sorghum, and pearl millet. (10–12). The two species differ in their ability to utilize carbon compounds for their growth. While *A. lipoferum* can utilize glucose, *A. brasilense* fails to do so (13). *A. brasilense* grows well on TCA cycle intermediates such as succinate or malate as a carbon source. It also has the ability to utilize fructose, galactose, gluconate, and l-arabinose but not glucose (13–17). Of these sugars, the fructose transport system has been studied in greater detail (15, 16, 18). Fructose was shown to favor the formation of flocs, melanin, and cysts in *A. brasilense* Sp7 (19–21).

In many proteobacteria such as *E. coli* and *Pseudomonas putida*, FruA, FruB, and FruK are encoded in a single operon (*fru* operon) that is responsible for the transport and phosphorylation of fructose (PTS^Fru^) and involves two polyproteins (2). One of these polyproteins FruB consists of a soluble EIIA-M-HPr fusion. The second, FruA, which is membrane-bound, includes the permease domains EIIB-EIIC. The genome of *A. brasilense* Sp7, also encodes a PTS^Fru^ which consists of two polyproteins. Although the membrane-bound FruA consists of EIIB and EIIC domains, the cytosolic FruB is a larger polyprotein and consists of EIIA-HPr-EI domains (15). Fructose-1-phosphate, the product of PTS^Fru^ reactions, enters the glycolytic pathway for further catabolism after its conversion to fructose-1,6-bisphosphate by 1-phosphofructokinase (1-PFK) (16, 17). All three enzymes, such as enzyme I (FruB), enzyme II (FruA), and 1-PFK (FruK), are induced by fructose in *A. brasilense*. We have recently shown that fructose enables *A. brasilense* to co-metabolize ethanol by inducing the PQQ-dependent enzymes associated with ethanol catabolism (22). Although biochemical and physiological studies involving the kinetics of enzymes involved in fructose transport in *A. brasilense* have been investigated in greater detail (18), analysis of the genes involved, and their regulation was not known. The aim of this study was to understand the role of some of the proteins induced by fructose in *A. brasilense* Sp7. Here, we have identified fructose inducible proteins, and investigated the function of proteins involved in making PTS^Fru^, a Type 6 secretion system (T6SS) and chemotaxis in *A. brasilense* Sp7.

## RESULTS

### Identification of fructose inducible proteins in *A. brasilense* Sp7

In order to identify the proteins expressed in *A. brasilense* Sp7 when it is grown with fructose as sole source of carbon, we compared the proteome of the exponential phase cultures of *A. brasilense* Sp7 (1.0 OD_600_ having about 5 × 10^8^ cells/mL) supplemented with 40 mM fructose or 40 mM malate as sole source of carbon. Proteomic analysis showed that a total of 715 proteins were upregulated in fructose-grown cultures, out of which 576 were upregulated by >2-fold, whereas 139 were detected in fructose-grown cultures only (see Table S1 in the supplemental material). Based on the gene IDs, we tried to identify the upregulated proteins that are encoded in the vicinity of each other as a part of an operon that may probably be involved in a specific pathway or perform a common function (Table 1). Among the upregulated proteins, we found FruA, FruB, and FruK, which constitute a fructose phosphotransferase system (fructose-PTS). The FruK, FruB, and FruA proteins were upregulated by 12.01-, 44.51-, and 3.84-fold, respectively. Observation of conspicuously lower level of induction of FruA, a membrane protein, is likely to be due to the protein extraction protocol, which favors soluble proteins.

**TABLE 1:** Upregulated proteins that are part of an operon or that are involved in a specific pathway and perform a common function

Gene ID/KEGG ID	Gene description	Gene symbol	Gene function	Abundance ratio
AMK58_00980	Cytochrome c oxidase cbb3-type subunit I	*cytN*	Cytochrome C-oxidase	2.103
AMK58_00985	Cytochrome c oxidase cbb3-type subunit II	*cytO*		2.476
AMK58_00995	Cytochrome c oxidase cbb3-type subunit III	*ccoP*		2.392
AMK58_02380	Chemotaxis protein CheB	*cheB*	Chemotaxis proteins of Che4 system	2.776
AMK58_02395	Methyl-accepting chemotaxis protein	*MCP*		2.966
AMK58_02405	Chemotaxis protein CheA	*cheA*		2.768
AMK58_04810	Glycolate oxidase subunit GlcE	*glcE*	Glycolate oxidase	3.303
AMK58_04815	Glycolate oxidase iron-sulfur subunit	*glcF*		3.678
AMK58_06465	Pyrroloquinoline quinone biosynthesis peptide chaperone PqqD	*pqqD*	PQQ biosynthetic proteins	2.051
AMK58_06470	Pyrroloquinoline-quinone synthase PqqC	*pqqC*		4.06
AMK58_06475	Coenzyme PQQ synthesis protein B PqqB	*pqqB*		3.081
AMK58_07470	ABC transporter ATP-binding protein	*livG*	ABC transporter	2.233
AMK58_07480	ABC transporter substrate-binding protein	*livK*		18.761
AMK58_07525	Xanthine dehydrogenase	*xdhC*	Xanthine dehydrogenase	2.358
AMK58_07540	MoxR family ATPase	*moxR*		2.206
AMK58_07545	Xanthine dehydrogenase family protein subunit M	*coxM*		4.97
AMK58_07550	Xanthine dehydrogenase family molybdopterin-binding subunit	*coxL*		6.72
AMK58_07555	(2Fe-2S)-binding protein	*coxS*		4.393
AMK58_07560	TAXI family TRAP transporter solute-binding subunit			4.656
AMK58_18725	Molybdenum ABC transporter ATP-binding protein	*modC*	Molybdenum ABC transporter	2.191
AMK58_18730	Molybdenum transport system permease	*modB*		2.387
AMK58_18735	Molybdate ABC transporter substrate-binding protein	*modA*		2.248
AMK58_18740	Molybdenum-dependent transcriptional regulator	*modE*		2.061
AMK58_18765	Type VI secretion system protein TssA	*tssA*	Type VI secretion system	2.415
AMK58_18775	Type VI secretion system protein TssL	*tssL*		3.322
AMK58_18780	Type VI secretion system protein TssK	*tssK*		1.961
AMK58_18785	Type VI secretion system protein TssJ	*tssJ*		100
AMK58_18830	Type VI secretion system protein TssB	*tssB*		82.379
AMK58_18835	Type VI secretion system protein TssC	*tssC*		13.643
AMK58_18840	Type VI secretion system tube protein	*tss hcp*		36.97
AMK58_18850	Type VI secretion system protein TssF	*tssF*		1.901
AMK58_18860	Type VI secretion system protein VasG	*clpV*		2.96
AMK58_18865	Type VI secretion system secreted protein VgrG	*vgrG*		4.029
AMK58_19555	Chemotaxis protein CheR	*cheR*	Chemotaxis proteins of Che1 system	2.757
AMK58_19565	Chemotaxis protein CheY	*cheY*		1.983
AMK58_19570	Chemotaxis protein CheW	*cheW*		2.532
AMK58_19575	Chemotaxis protein, sensor kinase CheA	*cheA*		2.34
AMK58_22840	Protein TonB	*tonB*	TonB system transporter	3.649
AMK58_22845	Biopolymer transport protein ExbD	*exbD*		17.055
AMK58_22850	Biopolymer transport protein ExbB	*exbB*		7.045
AMK58_23610	Chemotaxis family, sensor kinase CheA	*cheA*	Chemotaxis proteins of Che2 system	2.181
AMK58_23635	Purine-binding chemotaxis protein CheW	*cheW*		2.423
AMK58_25875	Fructose PTS system EIIBC or EIIC component	*fruA*	Fructose phosphotransferase system	3.84
AMK58_25880	1-phosphofructokinase	*fruK*		12.018
AMK58_25885	PTS fructose transporter subunit IIA	*fruB*		44.516
AMK58_27655	Tripartite tricarboxylate transporter substrate binding protein	*tctC*	Tripartite tricarboxylate transporter	8.151
AMK58_27660	Tripartite tricarboxylate transporter substrate binding protein	*tctC*		37.907
AMK58_09385	Flagellar motor protein MotA	*motA*	Flagellar motor proteins	2.615
AMK58_12925	Flagellar motor protein MotB	*motB*		3.374
AMK58_02395	Methyl-accepting chemotaxis protein	*MCP*	Methyl accepting chemotaxis proteins	2.966
AMK58_04440	Methyl-accepting chemotaxis protein	*MCP*		2.042
AMK58_05230	Methyl-accepting chemotaxis protein	*MCP*		2.118
AMK58_09085	Methyl-accepting chemotaxis protein	*MCP*		5.646
AMK58_19980	Methyl-accepting chemotaxis protein	*MCP*		4.41
AMK58_21580	Methyl-accepting chemotaxis protein	*MCP*		1.974
AMK58_26645	Methyl-accepting chemotaxis protein	MCP		2.123

In addition, several proteins TssA, TssL, TssK, TssJ, TssB, TssC, TssHcp, TssF, VasG, and VgrG, which are known to constitute a T6SS were upregulated. We also found chemotaxis proteins, including CheR, CheA, CheW, and CheY of the Che1 system, CheB, MCP, and CheA of the Che4 system, and CheA and CheW of the Che2 system, a 28 kDa flagellin protein, the flagellar motor proteins MotA and MotB, and at least seven methyl-accepting chemotaxis proteins (Table 1).

We also found upregulation of three proteins of the cytochrome c-oxidase operon, two proteins of the glycolate oxidase operon, the PQQ biosynthesis protein PqqBCD, two ABC transporters, six proteins of the xanthine dehydrogenase operon, four proteins of the molybdenum ABC transporter operon, the TonB system transporters ExbB, ExbD, and TonB, and two tripartite tricarboxylate transporters (Table 1).

### *A. brasilense* Sp7 genome encodes two putative fructose phosphotransferase systems

**Fig 1:**
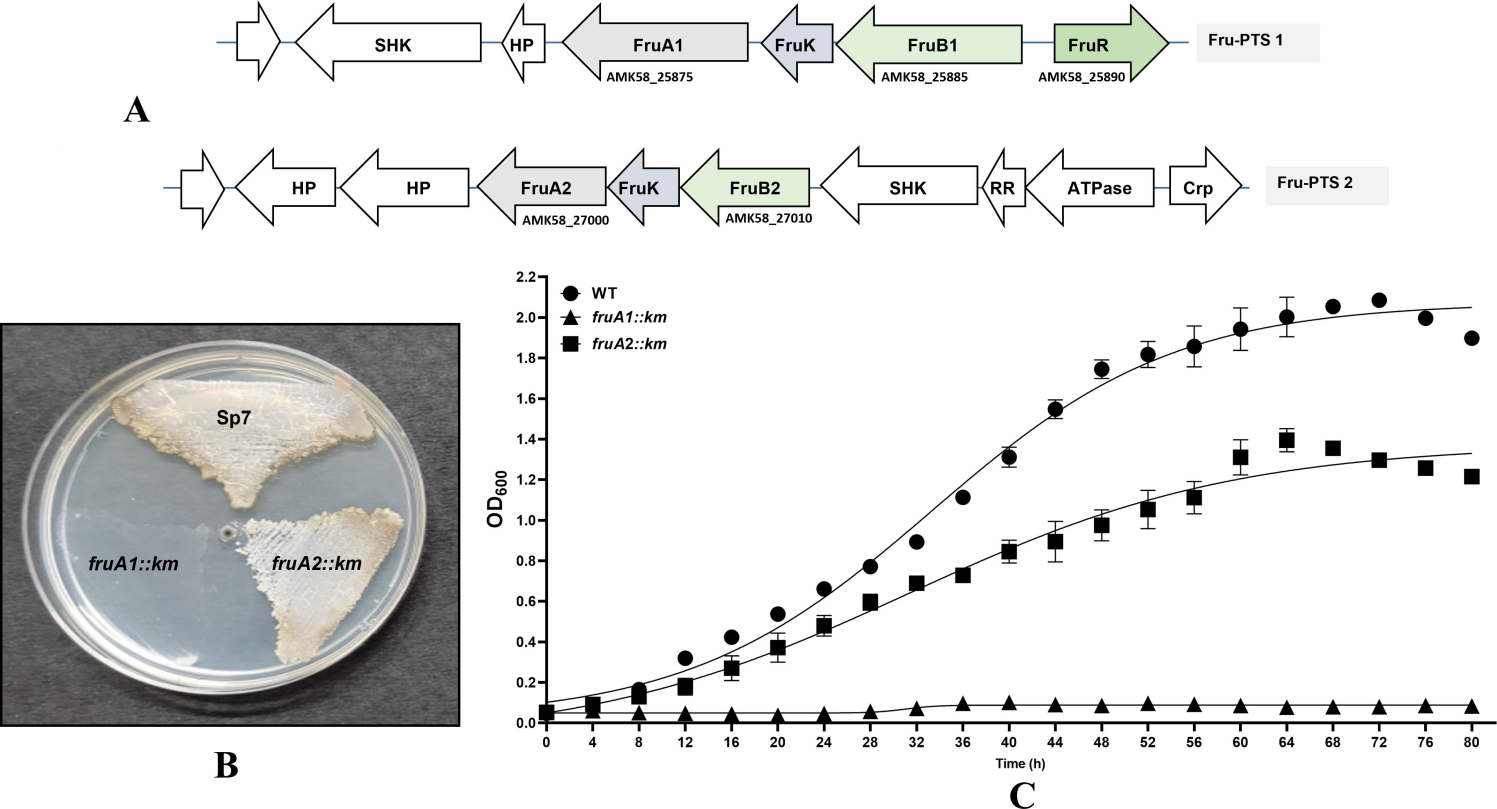
Organization of the genes encoding two putative fructose phosphotransferase systems (Fru-PTSs) in the genome of *Azospirillum brasiliense* Sp7. Each operon encodes FruB (EIIA-HPr-EI), FruK (Pfk), and FruA (EIIBC) proteins (**A**). The pointed arrows show the direction of the genes. ATPase, ATPase/histidine kinase; CRP, Crp/Fnr family transcriptional regulator; FruR, a LacI-type regulator; HP, hypothetical protein; RR, two-component system response regulator; SHK, sensor histidine kinase. Comparison of the growth of *A. brasilense* Sp7 (WT), *fruA1::km* and *fruA2::km* mutants on minimal medium agar plates (**B**) and in liquid minimal medium (**C**) containing 40 mM fructose as sole source of carbon (MFM). Each point on the curve shows mean value of the triplicates obtained from three independent experiments, and error bars at each point show standard deviation (SD).

Analysis of the proteome of *A. brasilense* Sp7 grown on fructose as sole carbon source showed upregulation of all the three components of a fructose PTS (designated as Fru-PTS1); FruA (AMK58_25875), FruK (AMK58_25880), and FruB (AMK58_25885). When we analyzed the organization of the genes encoding Fru-PTS using KEGG database (:AMK58_25885), we found an intergenic distance of 36n between *fru*A and *fru*K, and an overlap of 3n between *fru*K and *fru*B, suggesting that the three genes are organized in an operon, and FruK and FruB might be translationally coupled (Fig. 1A). A gene encoding FruR (AMK58_25890), a LacI-type regulator was located divergently to *fruB*. A closer examination of the genome of *A. brasilense* Sp7 revealed the annotation of an additional set of Fru-PTS genes (Fru-PTS2), which include genes encoding FruA (AMK58_27000), FruK (AMK58_27005), and FruB (AMK58_27010), organized as an operon. But, in place of a FruR homolog, a gene encoding a sensor histidine kinase (SHK) was located upstream of the *fruB* in the same orientation (Fig. 1A). The proteins corresponding to the second set of fructose PTS, however, were not upregulated by fructose. FruA, FruK, and FruB encoded from both the operons showed 55% (69%), 47% (60%), and 47% (61%) identities (similarities), respectively, between their amino acid sequences.

### Fru-PTS1 is the main PTS involved in fructose utilization

In order to identify the PTS involved in fructose utilization, we inactivated *fruA*, the last gene of both the *fru* operons by inserting a kanamycin resistance gene in the middle of the *fruA*. Growth of the *fruA1::km* and *fruA2::km* mutants was compared with that of *A. brasilense* Sp7 in the minimal medium with fructose as sole source of carbon. Figure 1B and C shows that inactivation of the *fruA1* led to a complete loss of the ability of the *fruA1::km* mutant to grow on fructose as sole carbon source suggesting its primary role in supporting growth on fructose. The growth of *fruA2::km* mutant, however, was significantly reduced but not completely inhibited (Fig. 1C) suggesting a secondary role of FruA2 in fructose utilization by *A. brasilense* Sp7.

### Complementation and cross-complementation of *fruA1::km* and *fruA2::km* mutants with *fruA1* show higher growth than the parent on fructose

**Fig 2:**
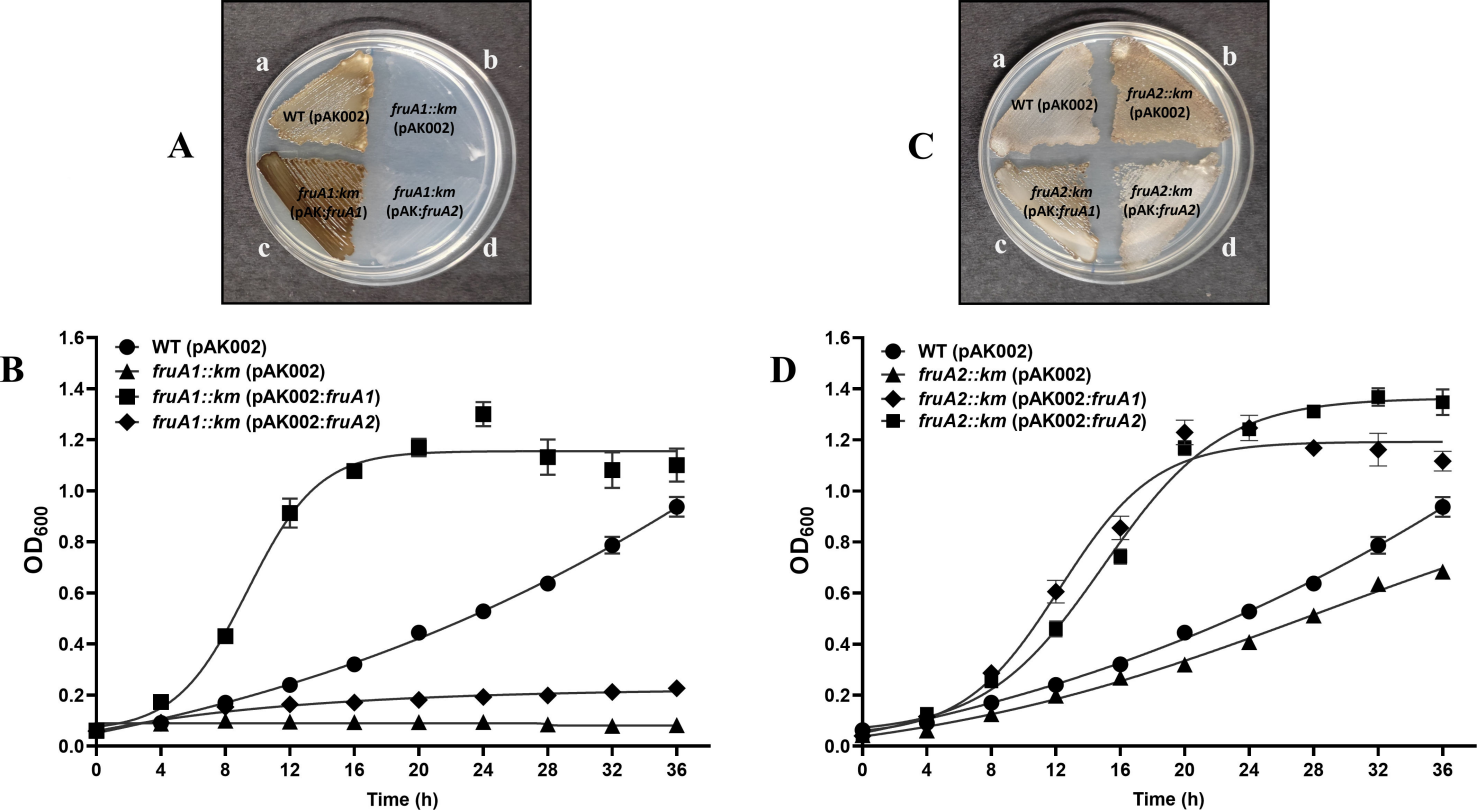
Comparison of the growth of the WT (pAK002), *fruA1::km* (pAK002)*, fruA1::km* (pAK002:*fruA1*), and *fruA1::km* (pAK002:*fruA2*) on MFM agar plates (**A**) and in MFM liquid minimal medium (**B**). Production of high amount of brown-pigmented melanin by (pAK002:*fruA1*) is notable (**A**). Comparison of the growth of WT (pAK002), *fruA2::km* (pAK002), *fruA2::km* (pAK002:*fruA1*), and *fruA2::km* (pAK002:*fruA2*) strains on MFM agar plates (**C**) and in MFM liquid medium (**D**). Each point on the curve shows mean value of the triplicates obtained from the three independent experiments, and error bars at each point show standard deviation (SD).

When we carried out experiments to examine the ability of *fruA*1, located on a broad host range vector, to complement the *fruA1::km* mutant, we found that the expression of *fruA1* led to a considerably faster and better growth (doubling time 187 min) than the parental strain Sp7 (doubling time 290 min). While complemented strain reached stationary phase within 16 h, *A. brasilense* Sp7 took longer than 36 h to reach the stationary phase. At 16 h, when OD_600_ of *A. brasilense* Sp7 was only around 0.25 (1.25 × 10^8^ cells/mL), the OD_600_ of the complemented strain reached to around 1.2 (6 × 10^8^ cells/mL) (Fig. 2B). Attempts to complement the *fruA1::km* mutant with *fruA2* gene showed very little restoration of growth (Fig. 2A and B). Although the growth of *fruA2::km* mutant was only slower than its parent, expression of *fruA1* as well as *fruA2* via broad host range plasmid improved the growth of *fruA2::km* mutant considerably (Fig. 2C and D). Overexpression of *fruA1* in *A. brasilense* Sp7 and its *fruA1::km* mutant (Fig. 2A) also led to the production of flocs in liquid medium and melanin-like pigment on agar plates.

### Overexpression of *fruA1* increases growth and fructose utilization ability of *A. brasilense* Sp7

**Fig 3:**
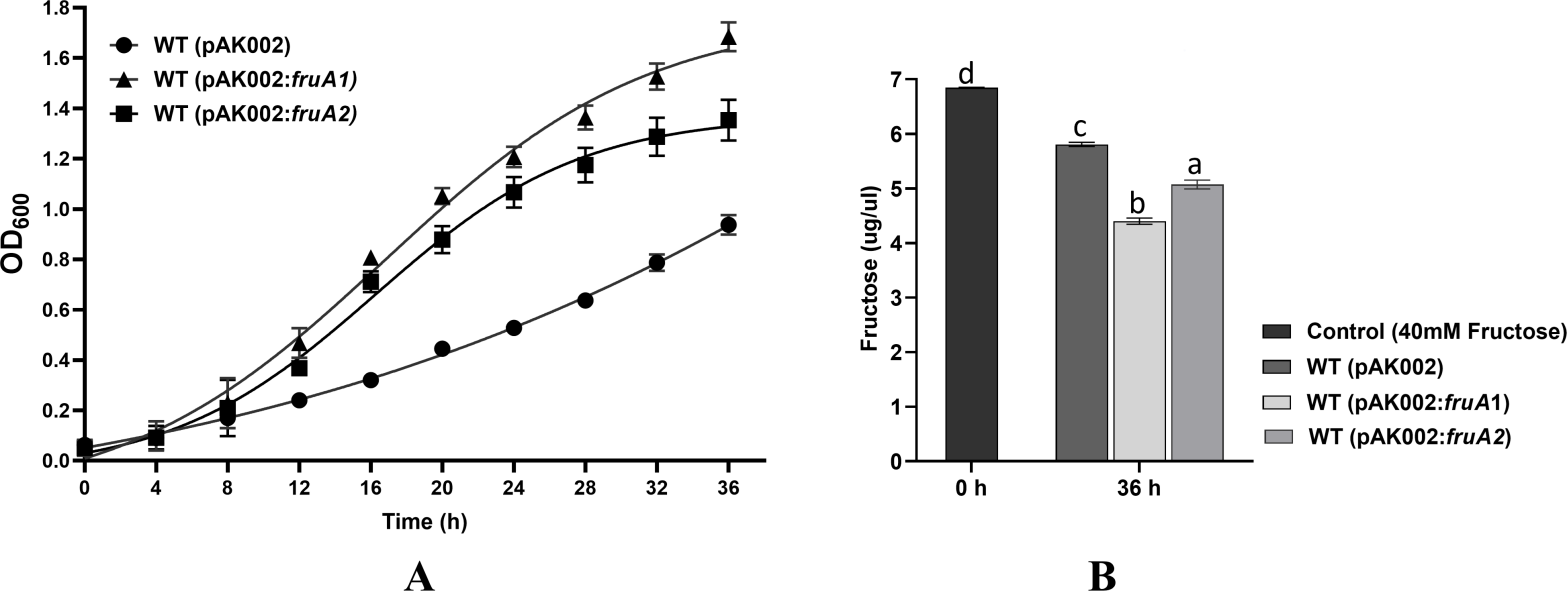
(**A**) Effect of over-expressions of *fruA1* or *fruA2* genes on the growth of *A. brasilense* Sp7 in MFM liquid medium. Each point on the curve shows the mean value of the triplicates obtained from three independent experiments and error bars at each point show standard deviation (SD). (**B**) Bar diagram showing residual fructose (µg/µL) in the spend culture medium (MFM) after growth of WT (pAK002), WT (pAK002:*fruA1*), and WT (pAK002:*fruA2*) strains of *A. brasilense* Sp7 for 36 h. Blank MFM without any inoculation was used as a control. Mean ± SD of triplicates from three independent experiments is indicated, and differences between mean values were compared. Lowercase letters above the bars represent different homologous subsets of different treatments after results of Duncan’s multiple comparison test, analyzed through SPSS software (different letters have *P* < 0.05).

Since overexpression of *fruA1* gene in the two *fruA::km* mutants resulted in the growth, which was better than that of their parent, we investigated the effect of overexpression of *fruA1* and *fruA2* genes on the growth of *A. brasilense* Sp7 on fructose as sole source of carbon, using two derivatives of the wild-type (WT) one overexpressing pAK002:*fruA1* and the other pAK002:*fruA2*. Figure 3A and Fig. S1 show that the overexpression of both *fruA1* and *fruA2* enhanced the growth of *A. brasiliense* Sp7, but the expression of *fruA1* shows relatively better growth of the host strain. We also observed that *A. brasilense* Sp7 and its mutants overexpressing *fruA1* reached stationary phase earlier than their parents and showed floc formation. A comparison of the residual fructose present after 36 h in the culture of *A. brasilense* Sp7 and its derivative overexpressing *fruA1* showed that the latter consumed more fructose (Fig. 3B).

### FruR is required for optimal growth in rich as well as minimal media

**Fig 4:**
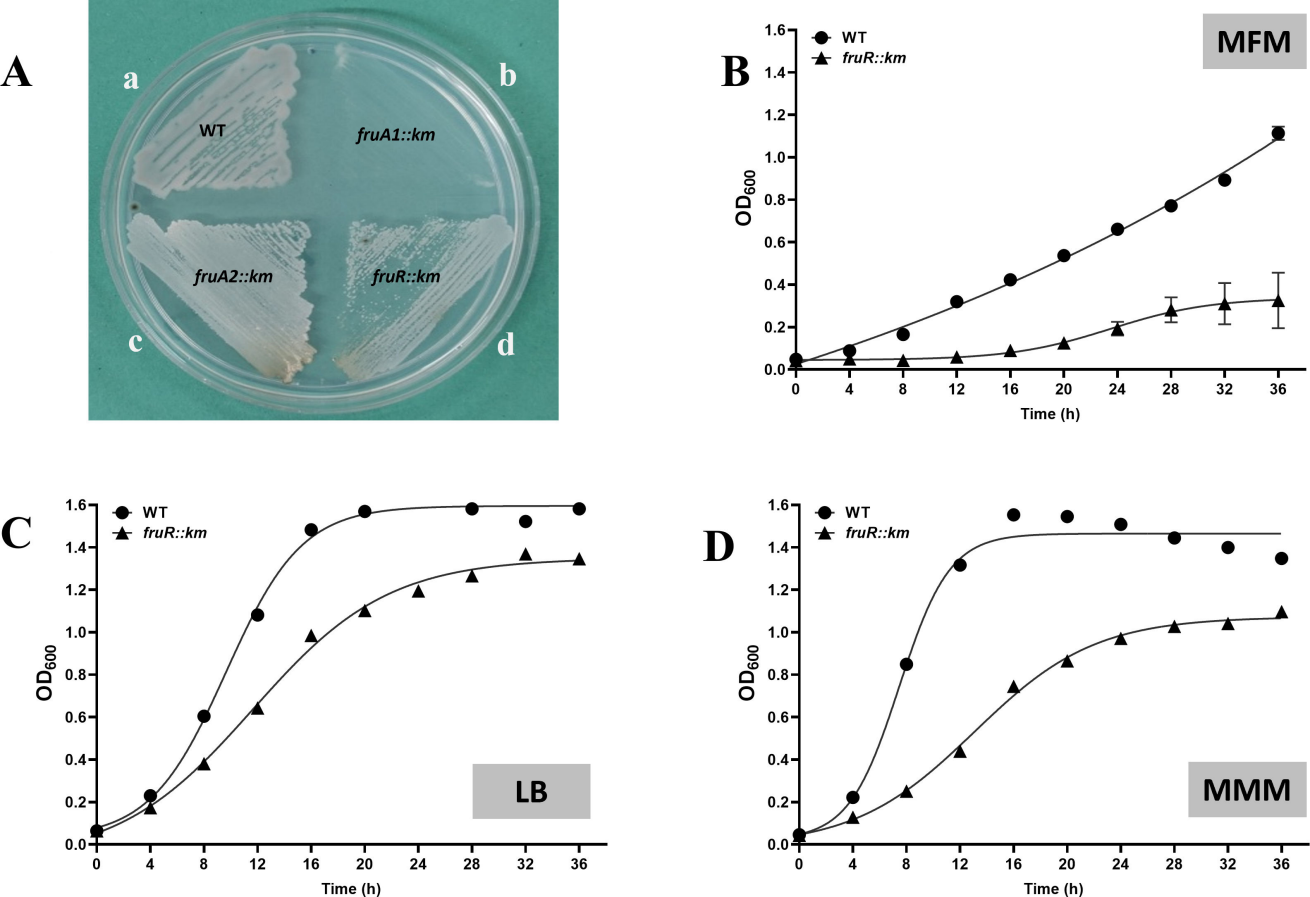
Comparison of the growth of *A. brasilense* Sp7 (WT), *fruA1::km* mutant, *fruA2::km* mutant, and *fruR::km* mutant on MFM agar plates (**A**). Growth curve of the WT and *fruR::km* mutant in MFM liquid medium (**B**),in MMM liquid medium (containing 40 mM malate as carbon source) (**D**), and in LB medium (**C**). Each point on the curve shows mean value of the triplicates obtained from three independent experiments, and error bars at each point show standard deviation (SD).

**Fig 5:**
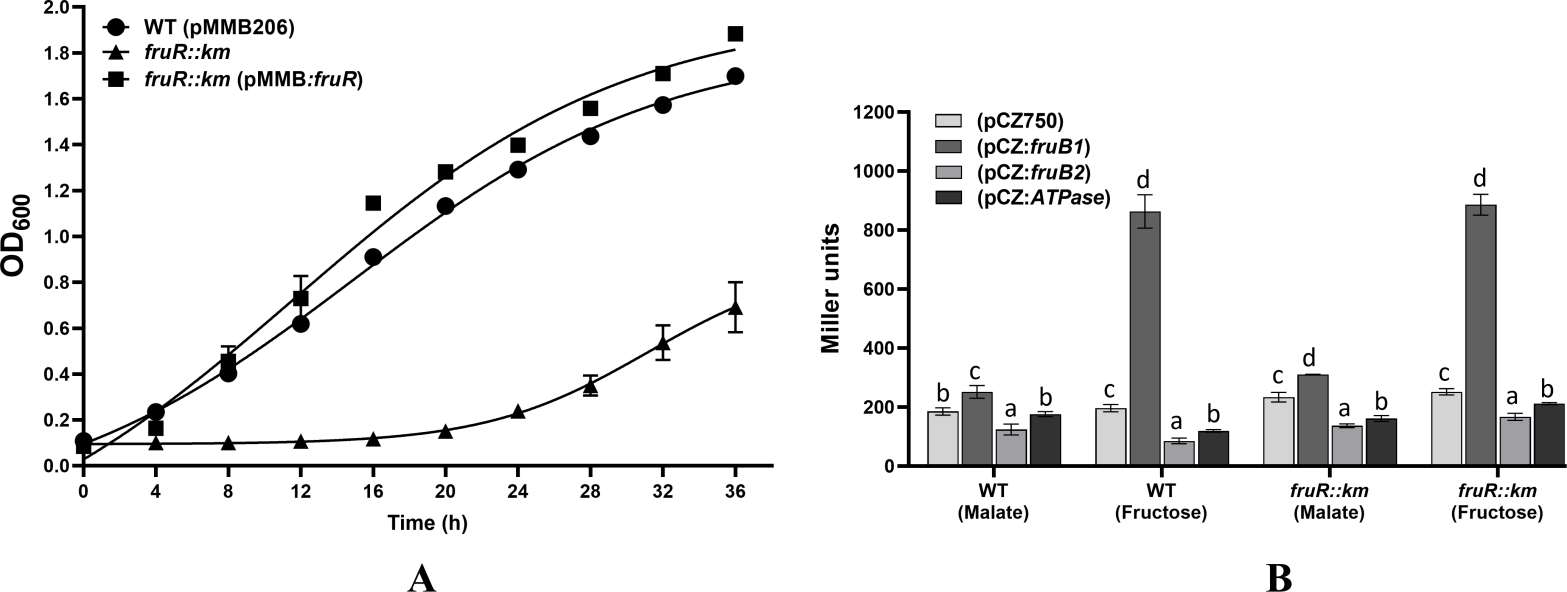
(**A**) Comparison of the growth of WT (pMMB206), *fruR::km* (pMMB206), and *fruR::km* (pMMB:*fruR*) in MFM liquid medium. Each point on the curve shows mean value of the triplicates obtained from three independent experiments, and error bars at each point show standard deviation (SD). (**B**) Bar diagram showing β-galactosidase activity from pCZ750 (plasmid vector control), *fruB1:lacZ, fruB2:lacZ*, and *ATPase:lacZ* fusion in WT (*A. brasilense* Sp7) and its *fruR::km* mutant grown in MMM or MFM for 6 h. Mean ± SD of triplicates from three independent experiments is indicated, and differences between the mean values were compared. Lowercase letters above the bars represent different homologous subsets of different treatments after results of Duncan’s multiple comparison test, analyzed through SPSS software (different letters have *P* < 0.05).

To elucidate the role of FruR, a LacI family transcription regulator, which was located divergently to the gene encoding FruB1, we inactivated the gene encoding FruR (AMK58_25890) and compared its growth with that of *A. brasilense* Sp7. Figure 4 shows that *fruR::km* mutant grew slower than *A. brasilense* Sp7 in minimal fructose medium (Fig. 4A and B), minimal malate medium (Fig. 4D), and LB medium (Fig. 4C). Since the growth of *fruR::km* mutant in fructose was considerably higher than that of *fruA1::km* (Fig. 4A) suggesting that FruR may not be involved in the regulation of the expression of *fruA1*. Expression of a cloned copy of the *fruR* located on pMMB206 in the *fruR::km* mutant shows complementation by restoration of the growth of *fruR::km* in the fructose medium (Fig. 5A).

### Expression of *fruB1* is not regulated by FruR

In order to examine the regulation of expression of *fruB1* and *fruB2*, we constructed *fruB1:lacZ* and *fruB2:lacZ* fusions. Since the distance between *fruB2* and the next gene located upstream was only 71 nucleotides, we were not sure whether *fruB2* was the first gene of the operon. Further, since the distance between the next two upstream genes was 17 and 9 nucleotides only (Fig. 1A), we hypothesized that the gene encoding ATPase could also be the first gene of the operon. Hence, we also constructed *ATPase:lacZ* fusion. All the three *lacZ* fusions were then mobilized in *A. brasilense* Sp7 and the *fruR::km* mutant. The effect of malate and fructose on the β-galactosidase activity showed that fructose was able to induce the expression of *fruB1:lacZ* fusion, whereas malate failed to do so (Fig. 5B). The *fruB2:lacZ* and *ATPase:lacZ* fusions containing the upstream intergenic regions of the genes encoding FruB2 and ATPase, the potential promoter of the Fru-PTS2, did not show any notable difference in the β-galactosidase activity showing the absence of any fructose-inducible promoter in their upstream region (Fig. 5B). We noted that the level of induction of the *fruB1:lacZ* fusion was nearly the same in *A. brasilense* Sp7 and *fruR::km* mutant (Fig. 5B), showing that FruR may not be required for the expression of *fruB1*.

### Determination of transcription start site of *fruB1*

**Fig 6:**
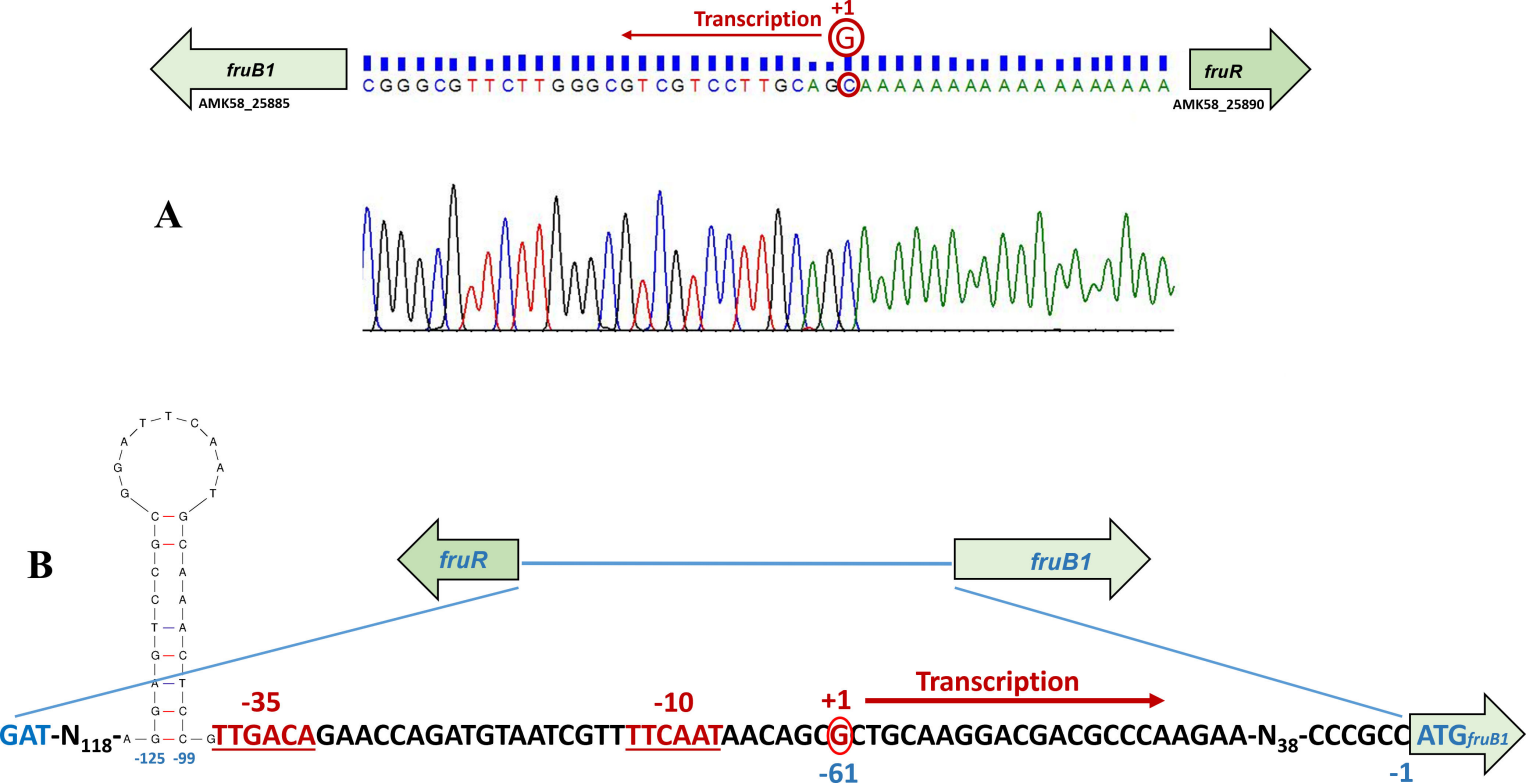
(**A**) Chromatogram showing the transcription start site of *fruB1*, determined by 5′ rapid amplification of cDNA ends (5′ RACE), in which “G,” complementary to the encircled C, is indicated as the transcription start point. The chromatogram sequence is in reverse complement form. (**B**) Nucleotide sequence of the intergenic region between the stop codon of *fruR* and the start codon of *fruB1* of *A. brasilense* Sp7, showing −10 and −35 hexamers of the promoter (red colored fonts and underlined), the location of the fifth stem loop and its position with respect to the first codon of *fruB1* is also indicated.

To identify the promoter elements of *fruB1*, we determined transcription start site (TSS) of *fruB1* using 5′ rapid amplification of cDNA ends (5′ RACE). The TSS of *fruB1* was a “G” located 61 nucleotides upstream from the start codon (ATG) of FruB1 ORF (Fig. 6A). Based on the TSS, the *fruB1* promoter contained TTCAAT as −10 motif and TTGACA as −35 motif, with a distance of 18 nucleotides between the two motifs (Fig. 6B).

### A stem-loop structure in the promoter upstream region of *fruB1* is involved in fructose inducibility and malate repression

**Fig 7:**
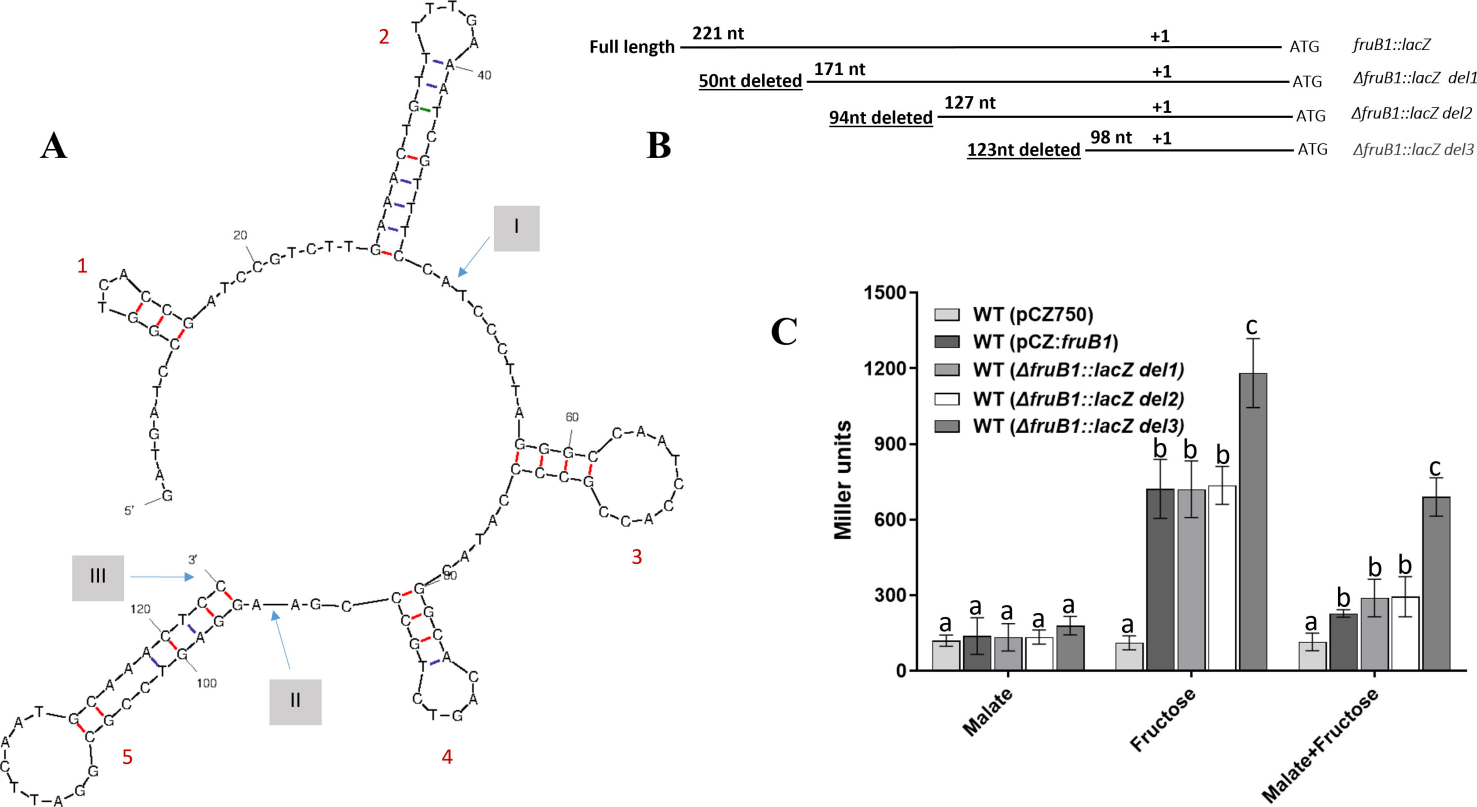
(**A**) Potential stem-loop structures predicted in the −221 to −99 bp upstream region of *fruB1* obtained by using the mfold tool (http://www.unafold.org/mfold.php). Numerals 1, 2, 3, 4, and 5 in red fonts indicate the stem-loop structures predicted in the −221 to −99 bp upstream of *fruB1* gene and labels I, II, and III show the location of the 5′ end points of the three deletions constructed in the *fruB1* promoter upstream region. The *fruB1::lacZ* contains the complete intergenic region between *fruB1* and *fruR*, *ΔfruB1::lacZ del1* lacks stem loops 1 and 2, *ΔfruB1::lacZ del2* lacks stem loops 1, 2, 3, and 4 and *ΔfruB1::lacZ del3* lacks all five stem loops including stem loop 5. (**B**) Graphical representation of *fruB1* upstream region and its three deletion derivatives fused with promoterless *lacZ* reporter, showing the number of deleted nucleotides (underlined) and the size of the remaining nucleotides in the three deletion derivatives, and the transcription start site (as +1). (**C**) The effect of malate (40 mM malate; MMM), fructose (40 mM fructose; MFM), and malate plus fructose (40 mM malate + 40 mM fructose) on the β-galactosidase activity of *A. brasilense* Sp7 harbouring pCZ750 (control), *fruB1:lacZ, ΔfruB1::lacZ del1, ΔfruB1::lacZ del2*, or *ΔfruB1::lacZ del3* fusions grown for 6 h. Mean ± SD of triplicates from three independent experiments is indicated, and differences between mean values compared. Lowercase letters above the bars represent different homologous subsets of different treatments after results of Duncan’s multiple comparison test, analyzed through SPSS software (different letters have *P* < 0.05).

The intergenic region between the start codons of *fruB1* and *fruR* consists of 221 bp. In order to identify presence of *cis*-acting regulatory elements in the promoter upstream region of *fruB1*, we constructed three deletion derivatives of the *fruB1* promoter upstream region with deletions of 50 bp (*del1*, −221 to −172), 94 bp (del2, −221 to −128), and 123 bp (del3, −221 to −99) after checking the presence of potential stem-loop structures in the 221 bp region using the mfold tool. Two potential stem-loop structures (1 and 2) were present in the 50 bp region from −221 to −172, two additional potential stem loops (3 and 4) in the 44 bp region from −171 to −128, and a fifth potential stem loop at −125 to −99 was present in the 29 bp region from −127 to −99. (Fig. 7A).

The full *fruB1*upstream region (221 bp) and its three deletion derivatives (*del1, del2*, and *del3*) were fused with a promoterless *lacZ* reporter (Fig. 7B), mobilized in *A. brasilense* Sp7 and assayed for their β-galactosidase activity after growing them in minimal medium containing malate, fructose, and malate plus fructose as carbon source. Figure 7C shows that *A. brasilense* Sp7 *fruB1* along with its 221 bp upstream region and three deletion derivatives showed fructose inducibility as β-galactosidase activity of fructose-grown cultures was several folds higher than that grown in malate. However, the β-galactosidase activity in case of *del3* was nearly two times that of the full length, *del1* and *del2*. When cultures were grown with fructose plus malate, we observed a distinct decline (40–70%) in the β-galactosidase activity in case of the full length as well as the three deletion derivatives. But in case of *del3* the β-galactosidase activity was least inhibited by malate, suggesting the presence of some important regulatory element in the 29 bp region located between −99 and −127 bp upstream of the start codon of *fruB1*. This suggested that the fifth stem-loop structure present at −125 to −99 in the 29 bp promoter proximal region could be the site for the binding of an unidentified repressor as well as an activator which might be responsible for induction by fructose and repression by malate.

### FruB1 and FruR are required for chemotaxis toward fructose

**Fig 8:**
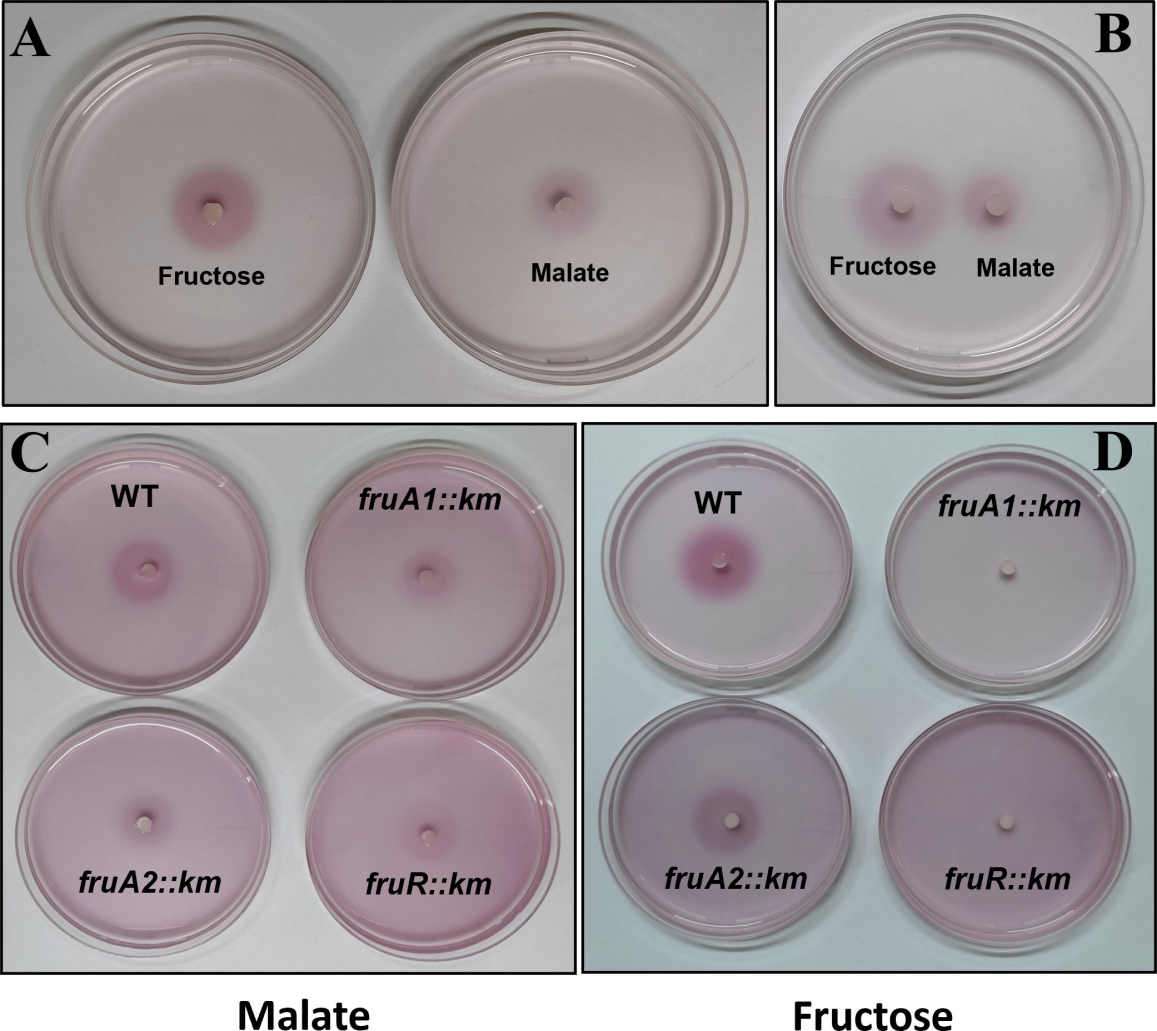
Comparison of chemotaxis of *A. brasilense* Sp7 (WT) toward malate and fructose in two different plates (**A**) and in a single plate (**B**). Chemotaxis of WT, *fruA1::km*, *fruA2::km*, and *fruR::km* mutants toward 1 M malate (**C**) or 1 M fructose (**D**) placed in the center of 0.3% MM agar containing a suspension of approximately 2.5 × 10^8^ cells/mL of the test strain and TTC (66 µg/mL).

Our proteomic data show that several proteins including a flagellin of 28 kDa, and proteins of chemotaxis systems Che1, Che2, and Che4 are upregulated in *A. brasilense* Sp7 by the supplementation of fructose. Keeping this in view, we compared chemotaxis of *A. brasilense* Sp7 toward malate and fructose by placing a drop (2 µL) each of 1 M fructose or malate solution in the center of the semi-solid minimal medium without any carbon source containing *A. brasilense* Sp7 cells. In order to improve visualization of *A. brasilense* cells, we included TTC in the semi-solid medium. The violet color observed in the plates indicates the location of *A. brasilense* cells. When cells exhibit chemotaxis toward the carbon compound located in the center, we observe increase in the size and intensity of the violet-colored ring indicating the extent of chemotactic response of *A. brasilense*. Figure 8A and B shows a relatively larger chemotactic ring of *A. brasilense* Sp7 around fructose suggesting that fructose might be a stronger chemoattractant than malate.

**Fig 9:**
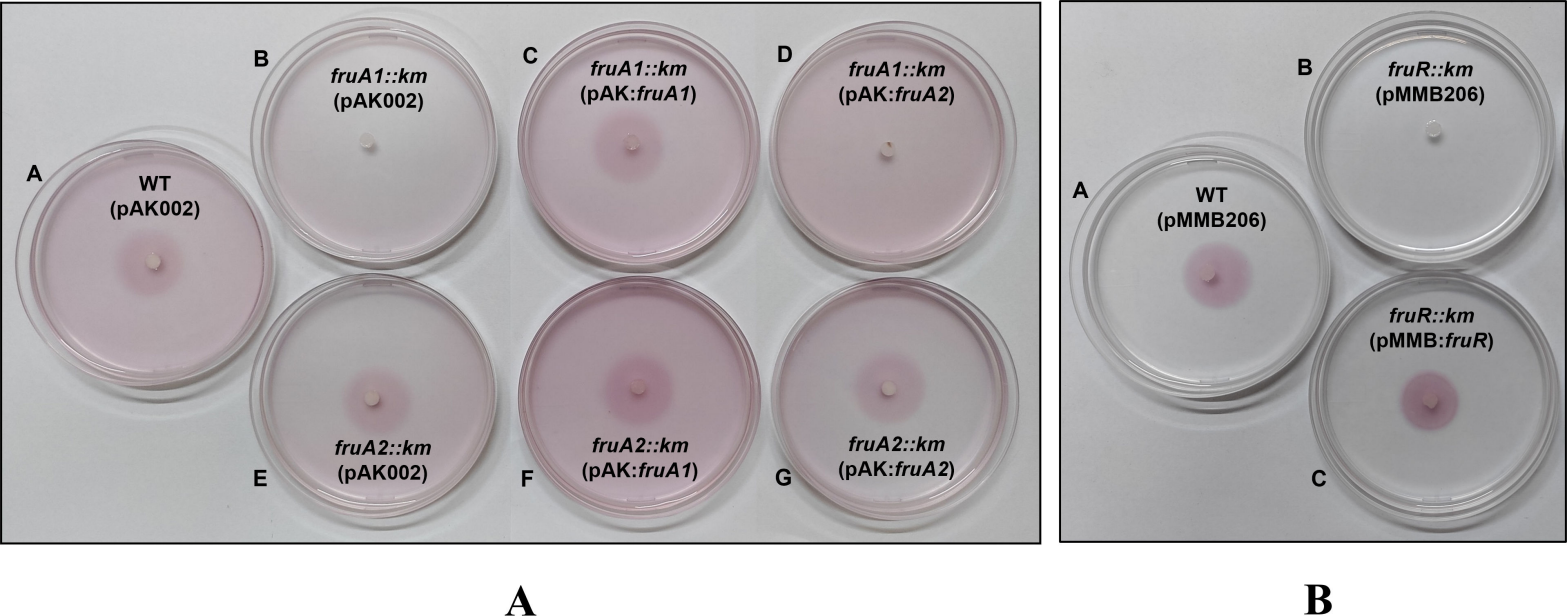
Comparison of chemotaxis of WT (pAK002), *fruA1::km* (pAK002)*, fruA1::km* (pAK002:*fruA1*), *fruA1::km* (pAK002:*fruA2*)*, fruA2::km* (pAK002), *fruA2::km* (pAK002:*fruA1*), and *fruA2::km* (pAK002:*fruA2*) (**A**), WT (pMMB206), *fruR::km* (pMMB206), and *fruAR::km* (pMMB:*fruR*) (**B**) toward 1 M fructose placed in the center of 0.3% MM agar containing suspension of approximately 2.5 × 10^8^ cells/mL of the test strain and TTC (66 µg/mL). Any appearance of vertical lines can be attributed to the method of imaging where plates are placed over two overlapping white papers to create a single photograph.

We also compared chemotaxis of *fruA1::km*, *fruA2::km*, and *fruR::km* mutants with that of *A. brasilense* Sp7 toward malate (Fig. 8C) and fructose (Fig. 8D). In this assay, comparison of the intensity of the violet color (due to TTC) of the chemotactic rings of *A. brasilense* Sp7 shows that fructose induces stronger chemotactic response than malate. *A. brasilense* Sp7 and *fruA2::km* mutant displayed almost equal levels of chemotactic response toward malate or fructose. Both *fruA1::km* and *fruR::km* mutants showed equal levels of chemotaxis toward malate, but they failed to do so toward fructose. Furthermore, we also examined the complementation and cross-complementation of the mutants to restore the presence of chemotactic rings toward fructose (Fig. 9A). Complementation with *fruA1* was able to restore (Fig. 9A, plate C), whereas cross-complementation with *fruA*2 was not able to restore the chemotactic ring *of fruA1::km* mutant (Fig. 9A, plate D) toward fructose. The complemented strain of the *fruR::km* mutant was also able to restore chemotactic ring formation similar to the parent (Fig. 9B).

### Fructose induces contact-dependent killing of *E. coli* by *A. brasilense* Sp7

**Fig 10:**
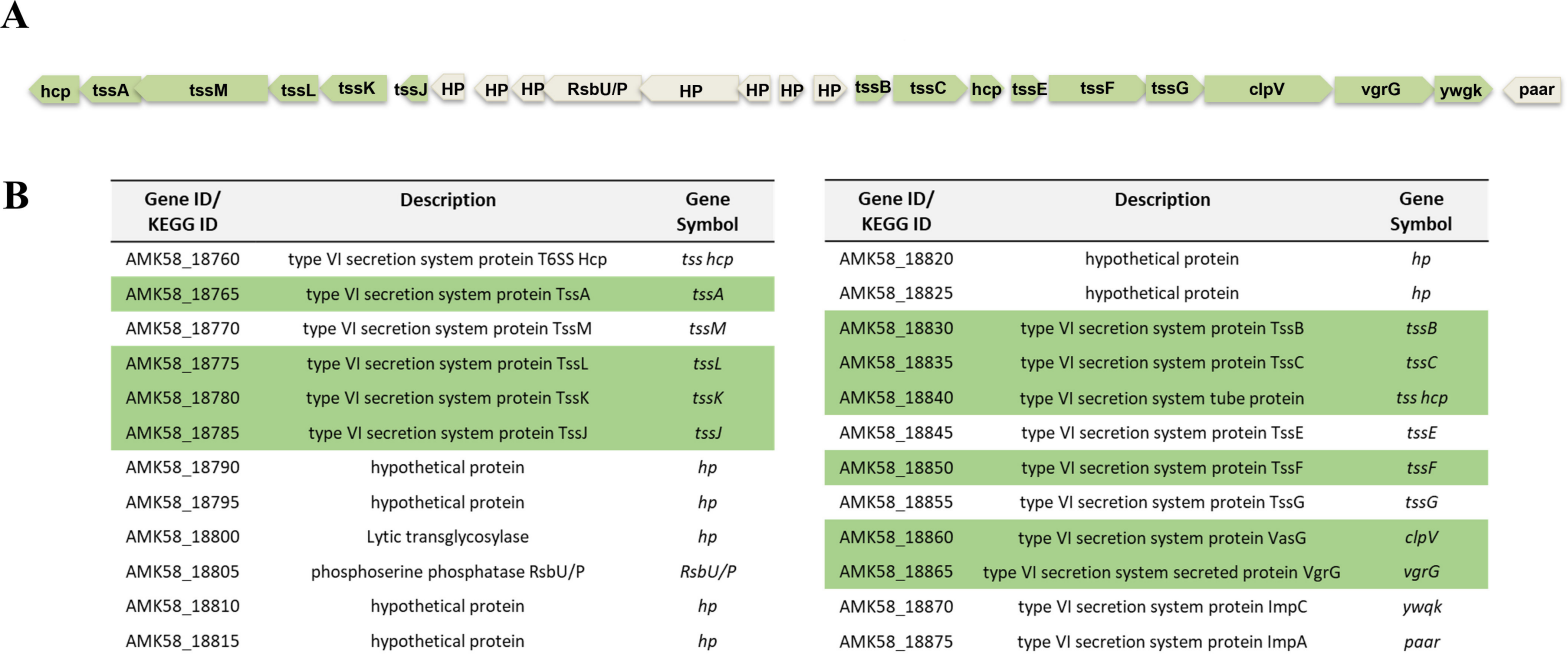
(**A**) Genetic organization of the Type 6 secretion system (T6SS) gene cluster in *A. brasilense* Sp7. (**B**) List of genes involved in the biogenesis of T6SS of *A. brasilense* Sp7 and the TSS genes upregulated by fructose in *A. brasilense* Sp7 (highlighted in green).

**Fig 11:**
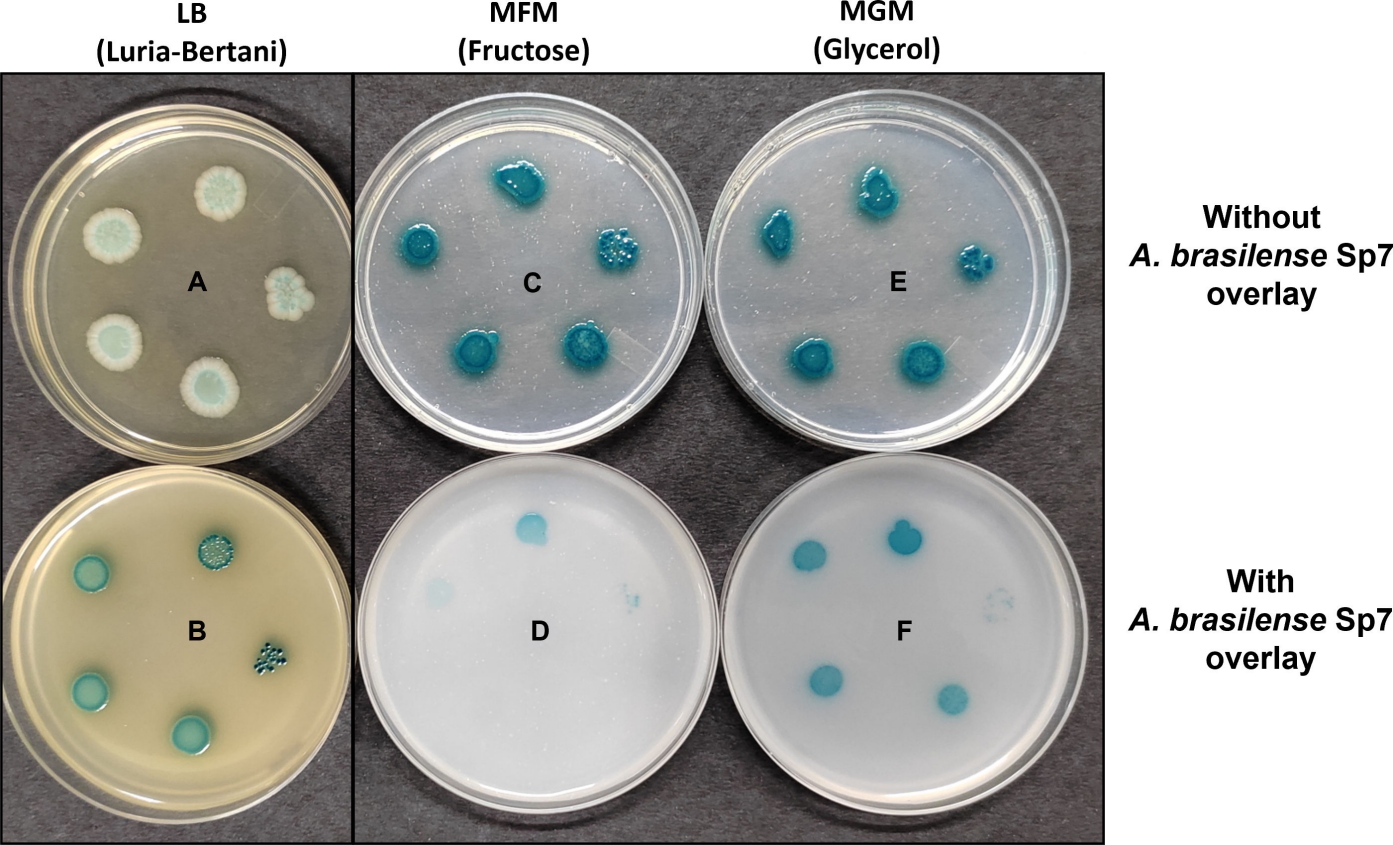
Fructose-induced contact-dependent inhibition of *E. coli* S17-1 by *A. brasilense* Sp7. Plates A, C, and E show growth of *E. coli* S17-1 (pAK002:*lacZ*) on LB, MFM, and MGM agar plates covered with soft agar without *A. brasilense* Sp7, respectively. Plates B (LB) and F (MGM) show growth, while plate D (MFM) shows inhibition of *E. coli* S17-1 (pAK002:*lacZ*) under similar conditions when they were overlaid with soft agar with *A. brasilense* Sp7 containing approximately 1 × 10^8^ cells/mL. MFM and MGM plates contain 40 mM fructose or 40 mM glycerol as the sole carbon source, respectively, and are supplemented with thiamine and proline. Blue colonies on plates show growth of different dilutions of 5 µL culture containing approximately 8 × 10^4^, 4 × 10^4^, 2 × 10^4^, 1 × 10^4^, and 0.5 × 10^4^ cells/mL of *E. coli* S17-1 (pAK002:*lacZ*), spotted (anti-clockwise) on an overlay of soft agar, and plates were observed after 4 days of incubation at 30°C.

Our proteomic data show that several proteins that constitute T6SS were upregulated by fructose. These proteins were encoded by genes that were part of a large cluster of genes (an island) involved in the biogenesis of T6SS (Fig. 10). In order to examine if *A. brasilense* Sp7 possesses the T6SS-mediated ability to kill other bacteria, we used *E. coli* as the target. For this we made a genetic modification in *E.coli*. We PCR amplified a promoterless β-galactosidase gene from *E. coli* BL21 (DE3), cloned it downstream of a constitutive kanamycin promoter in pAK002 (pAK002:*lacZ*) and transformed it into *E. coli* S17-1. This strain expresses β-galactosidase even in the absence of lactose or IPTG. The LB plate (plates A and B) and minimal medium plates containing glycerol (MGM) as sole carbon source (plates E and F) containing X-gal were covered with soft agar without or with *A. brasilense* Sp7. We then spotted serial dilution of *E. coli* S17-1 (pAK002:*lacZ*) culture on top of the soft agar overlay (Fig. 11). After 4 days, the blue-colored colonies of *E. coli* S17-1 (pAK002:*lacZ*) appeared on plates with soft agar overlay lacking or containing *A. brasilense* cells (plates A, B, E, and F). But when a similar experiment was done on minimal medium plates containing fructose as sole carbon source (plates C and D), growth of *E. coli* S17-1(pAK002:*lacZ*) was completely inhibited only on plate D, which is a minimal fructose plate with a soft agar overlay containing *A. brasilense*, showing contact-dependent killing of *E. coli* S17-1 (pAK002:*lacZ*) by *A. brasilense* Sp7. We also performed the same experiment on minimal medium plates containing fructose with different concentrations, including , 10, and 20 mM fructose as the sole carbon source, which also showed contact-dependent killing of *E. coli* S17-1 (pAK002:*lacZ*) by *A. brasilense* Sp7 (Fig. S2).

### T6SS mediates the ability of *A. brasilense* Sp7 to inhibit growth of *E. coli* and *A. tumefaciens*

**Fig 12:**
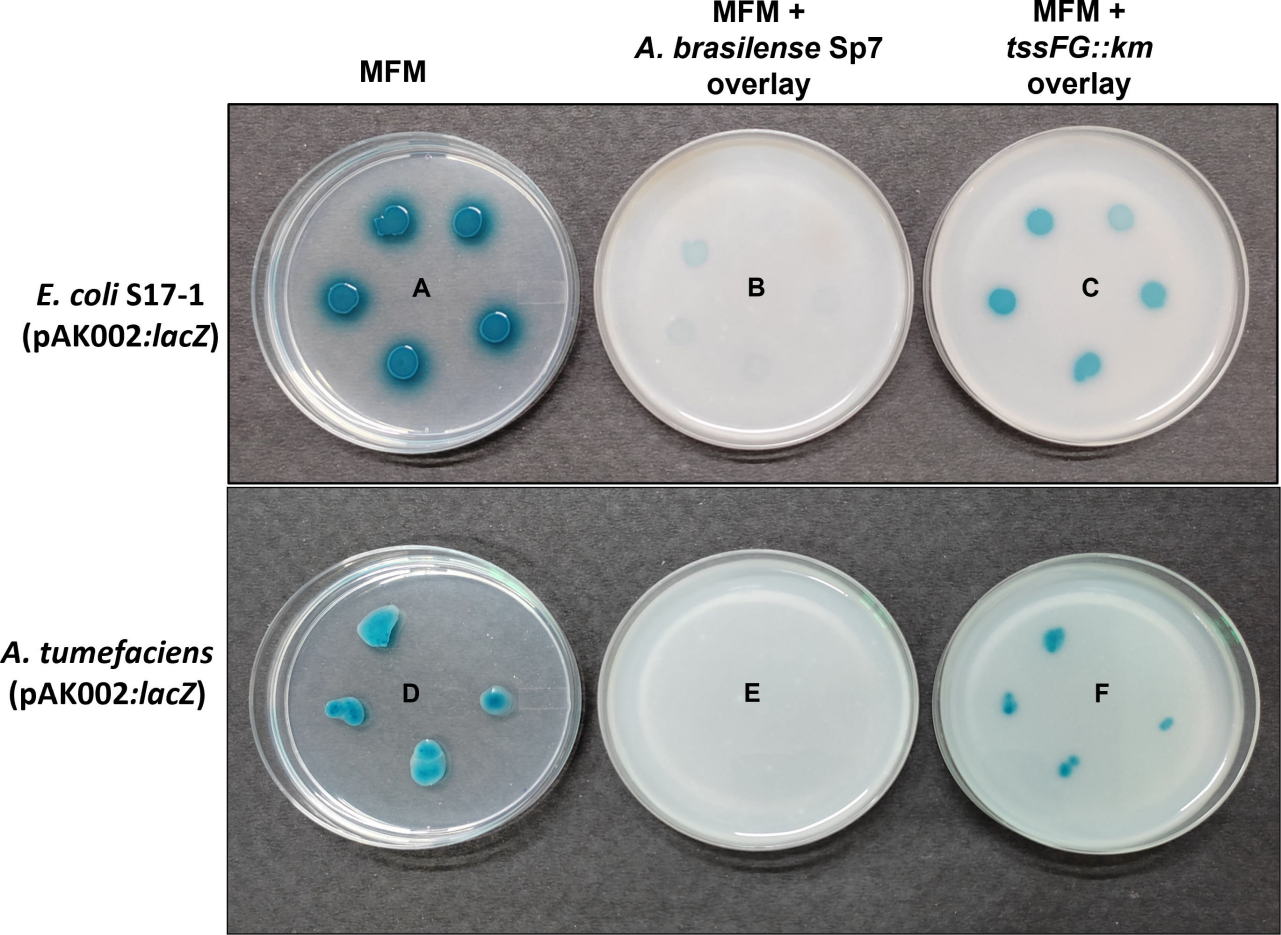
Role of T6SS in fructose-induced inhibition of *E. coli* S17-1 and *A. tumefaciens* EHA105 by *A. brasilense* Sp7. Plates A and D show growth of *E. coli* S17-1 (pAK002:*lacZ*) and *A. tumefaciens* EHA105 (pAK002:*lacZ*), respectively, on MFM plates. Plates B and E show the inhibition of growth of *E. coli* S17-1 (pAK002:*lacZ*) and *A. tumefaciens* EHA105 (pAK002:*lacZ*), respectively, when they were overlayed with soft agar containing 40 mM fructose along with approximately 1 × 10^8^ cells/mL of *A. brasilense* Sp7. Plates C and F show the growth of *E. coli* S17-1(pAK002:*lacZ*) and *A. tumefaciens* EHA105, respectively, under the same conditions when they were overlayed with soft agar containing 40 mM fructose along with approximately 1 × 10^8^ cells/mL of *tssFG::km* mutant. Blue colonies on the plates show growth of different dilutions of *E. coli* S17-1 (pAK002:*lacZ*) culture, containing approximately 8 × 10^4^, 4 × 10^4^, 2 × 10^4^, 1 × 10^4^, and 0.5 × 10^4^ cells/mL and *A. tumefaciens* EHA105 (pAK002:*lacZ*) culture, containing approximately 1 × 10^2^, 0.5 × 10^2^, 0.25 × 10^2^, 0.125 × 10^2^, and 0.625 × 10^1^ cells/mL spotted (anti-clockwise) on an overlay of soft agar, and plates were observed after 4 days of incubation at 30°C.

In order to examine if the T6SS of *A. brasilense* Sp7 was responsible for the killing of *E. coli* S17-1 cells, we inactivated T6SS by inserting a kanamycin resistance gene in between *tssF* and *tssG* genes to construct a *tssFG::km* mutant (Fig. 10A). As per genome annotation, 36 nucleotides of the gene encoding N-terminal part of TssG (AMK58_18855) overlap with the C-terminal part of the gene encoding TssF (AMK58_18850). These two proteins are essential components of the T6SS, which constitute an important part of the baseplate complex (23, 24). The WT copy of the *tssFG* genes in *A. brasilense* Sp7 was replaced by the mutated copy (*tssFG::km*) via homologous recombination. The insertion of the mutated copy of the gene (*tssFG::km*) was confirmed by PCR using appropriate combinations of primers. After confirming the genotype of the mutant, we compared the ability of the *tssFG::km* mutant to kill *E. coli* S17-1 and *A. tumefaciens*. Figure 12 shows that although *A. brasilense* Sp7 inhibited the growth of *E. coli* S17-1 and *A. tumefaciens* in the presence of fructose, the ability of the *tssFG::km* mutant to exhibit similar growth inhibitory effect was highly compromised indicating that a functional T6SS is required for optimal killing of *E. coli* S 17-1 and *A. tumefaciens* cells.

## DISCUSSION

In this study, we have identified fructose-inducible proteins which showed that out of the two putative fructose PTS encoded by *A. brasilense* Sp7 genome, PTS1 plays a major role in fructose utilization. We also noted that fructose is able to induce the expression of a large number of methyl-accepting chemotaxis proteins which are receptors for different ligand molecules to trigger chemotaxis (25, 26). Upregulation of multiple proteins of Che1, Che2, and Che4 chemotaxis systems (27) and MotA, MotB, and a 28 kDa flagellin indicated that fructose might have a profound effect on chemotaxis by *A. brasilense* Sp7. This observation was also corroborated by a relatively stronger chemotactic response of *A. brasilense* Sp7 to fructose than malate. Fructose also upregulated the expression of several ABC transporters including those for amino acids, branched-chain amino acids, peptides, sugar, cobalt, molybdenum, sulfate, and nitrate, TRAP transporters, C4 dicarboxylate transporter, tripartite tricarboxylate transporters, and TonB-dependent transporters of Iron. In order to uptake and utilize fructose, three essential component proteins of the fructose-PTS were upregulated. We have also found that the proteins involved in PQQ synthesis and proteins dependent on PQQ for their activity, such as alcohol dehydrogenases were upregulated by fructose.

When we found an additional set of genes encoding a fructose-PTS2 in the genome *A. brasilense* Sp7, we investigated the role of the two PTS in fructose utilization by inactivating *fruA* gene of each PTS, which encodes the membrane-bound EIIBC protein involved in the binding and transport of fructose. We found that fructose-PTS1 was the main PTS involved in the transport of fructose. The Fru-PTS2 is neither inducible by fructose nor seems to be specific for the transport of fructose. It may possibly be involved in the transport of some other sugars but might also transport fructose. In *E. coli*, there are several Fru family PTS systems but only one is involved in fructose transport (4). The observation that complementation of the *fruA1::km* mutant by plasmid borne-*fruA1* gene led to a higher growth of the complemented strain on fructose than that of fructose-induced *A. brasilense* Sp7 suggested that induction by fructose may not produce adequate amount of fructose-specific EIIBC transporters (FruA) in the membrane for the maximum growth of *A. brasilense* Sp7. To validate this assumption, we compared the growth of *A. brasilense* Sp7 with its derivative overexpressing *fruA1* gene, which showed a higher growth of the latter over the former on fructose. This was corroborated by the lower level of fructose in the spent medium of the culture of *A brasilense* Sp7 overexpressing *fruA1*. An increased growth of parent also by the overexpression of FruA2 further suggests that the amount of FruA1 protein produced naturally by induction of *A. brasilense* by fructose is limiting. This is why overexpression of FruA1 or FruA2 in *A. brasilense* results in improved growth of fructose.

These observations suggested that the copy number of the FruA (EIIBC) protein present in the inner membrane of *A. brasilense* Sp7 may not be enough to exhibit its maximal growth potential on fructose. And hence, overexpression of FruA via plasmid might be responsible for an increased number of EIIBC components in the membrane leading to an enhanced uptake and utilization of fructose for growth. Since *A. brasilense* Sp7 overexpressing *fruA1* attained stationary phase sooner than its parent *A. brasilense* Sp7, it also formed aggregates in liquid medium and produced copious amount of melanin-like brown black pigment on solid or semi-solid agar plates earlier than the WT. *A. brasilense* is known to form aggregates/flocs (19) in the liquid medium and to produce brown-black pigmentation and cysts during stationary phase when it is grown on solid agar plates containing 8 mM fructose and 0.5 mM KNO_3_ or 37 mM fructose and 4 mM NH_4_Cl (20, 21), but media containing malate or succinate as carbon source do not cause flocculation. It seems that the presence of fructose signals an unfavorable situation for *A. brasilense* as it responds by producing flocs, cysts, and melanin to prepare itself for an adverse stressful situation.

Like many other bacteria, the gene encoding FruR is organized divergently to the *fruBKA* operon in *A. brasilense* Sp7 (28). Inactivation of *fruR* gene in *A. brasilense* Sp7 led to a conspicuous decline in the growth of the *fruR::km* mutant, but in comparison to the *fruA1::km* mutant, which did not grow at all on fructose, the growth of *fruR::km* mutant was notably higher. This indicated that FruR does not act as an activator of the expression of the *fruBKA* operon in *A. brasilense*. If the expression of *fruBKA* operon is under the positive control of FruR, the growth of *fruR::km* mutant on fructose should have been at par or lower than the *fruA::km* mutant. Further, if the expression of *fruBKA* operon is under the control of FruR, the activity of *fruB1:lacZ* fusion should have declined in the *fruR::km* mutant. But, high level of fructose inducible expression of *fruB1:lacZ* fusion in *A. brasilense* Sp7 as well as in the *fruR::km* mutant indicated that expression of *fruBKA* operon was not dependent upon FruR. The slow growth of *fruR::km* mutant even on LB and malate suggested that FruR is a pleiotropic regulator and not just a fructose-specific regulator. This observation was further corroborated by equally the high level of induction of *fruB1:lacZ* fusion by fructose in *A. brasilense* Sp7 and its *fruR::km* mutant. However, it was intriguing to note that *fruR::km* mutant, like *fruA1::km* mutant, failed to exhibit chemotaxis toward fructose. The inability of *fruR::km* mutant to show chemotaxis toward fructose suggests that it might be involved in chemosensing and chemotaxis. In *P. putida*, FruR acts as a repressor which binds to the operator located upstream of *P_fruB_* promoter and represses *fruBKA* operon and fructose utilization (28). In *E. coli*, however, in addition to repressing the *fruBKA* operon, FruR is an activator of gluconeogenic carbon sources and hence has been renamed Cra (6).

Since malate is preferred over fructose as a carbon source by *A. brasilense* it exerts malate repression in a manner similar to the “catabolite repression” exerted by glucose in *E. coli* and succinate in *Pseudomonas*. Here, we noticed that the induction of *fruB1::lacZ* fusion by fructose was inhibited in the presence of malate (Fig. 7C), we anticipated that the *cis* elements responsible for the induction by fructose and repression by malate (a dicarboxylate) might be located in the upstream region of the *fruB1* promoter. In order to identify such elements, we constructed three deletion derivatives of the *fruB* upstream region and examined their inducibility by fructose and repressibility by malate. The first two deletions behaved similarly to the full-length upstream region, but the third deletion derivative not only showed considerably higher induction as compared to the other three, but malate addition could not repress the induction fully. Analysis of the secondary structures in the *fruB1-fruR* intergenic region showed that although the first and second deletions harbored two stem-loop structures each, they are distal to *fruB1* and proximal to *fruR*, and hence may not affect the expression of *fruB1*. However, the DNA that has been deleted in *del3* seems to play an important role in its induction by fructose and repression by malate. Hence, we identified a potential regulatory region located between −99 and −125 bp upstream of AUG. In addition, there appears to be another regulatory region responsible for the induction of *fruB1* by fructose in the region downstream of −99. Since the −35 sequence of the mapped *fruB1* promoter is immediately downstream to −99 and the stem loop 5, this implies that the activating sequence might either overlap with the promoter or located downstream of the +1. Classically, the activators bind upstream of their cognate promoters and are known to enhance binding of RNA polymerase to the downstream promoter (29). Further, since malate caused >2-fold repression for full-length, *del1*, and *del2* fusions but <2-fold for *del3*, it appears that the site of repression might not be limited to −99 to −127 but require some sequences of the DNA region overlapping the −99 boundary.

Notable among the fructose upregulated proteins were several proteins that are involved in the making of a multiprotein machine, the T6SS (T6SS), which is deployed by numerous Gram-negative bacteria to deliver toxic effectors into both prokaryotic and eukaryotic cells (30, 31). A recent study on *A. brasilense* Az39 genome revealed that it contained two complete sets of genes encoding T6SS, including the T6SS1 that is induced by the indole-3-acetic acid (IAA) phytohormone (24). The *A. brasilense* T6SS provides antagonistic activities against a number of plant pathogens such as *Agrobacterium*, *Pectobacterium*, *Dickeya*, and *Ralstonia* species *in vitro*, suggesting that, in addition to promoting growth, *A. brasilense* might confer T6SS-dependent bio-control protection to plants against bacterial pathogens (24).

In this study, we have shown that the genome of *A. brasilense* Sp7 contains a single-gene cluster (T6SS1) which encodes a complete T6SS. Several of the proteins that constitute T6SS are induced by fructose in *A. brasilense* Sp7. Using competition assays, we have shown that it is able to kill *E. coli* and *A. tumefaciens* in a contact-dependent manner in the presence of fructose. Since the genome of *A. tumefaciens* does not encode any T6SS, its growth is also inhibited by *A. brasilense* Sp7 on fructose. This indicated that *A. brasilense* Sp7 produces a functional fructose-inducible T6SS-mediated anti-bacterial system that enables it to outcompete some of the rival plant-associated bacteria found in the rhizosphere (32). The presence of T6SS in *A. brasilense* Sp7 might be an additional factor responsible for its rhizocompetence and wide occurrence in the rhizosphere of a wide range of plants.

## MATERIALS AND METHODS

### Bacterial strains, plasmids, and chemicals

*A. brasilense* Sp7 and its mutants (*fruA1::km*, *fruA2::km*, *fruR::km*, and *tssFG::km*) were maintained at 30°C on minimal medium agar plates supplemented with malate (40 mM) as sole source of carbon (33). *A. tumefaciens* EHA105 was maintained at 30°C and *E. coli* strains DH5α, S17-1, and BL21 (DE3) were maintained at 37°C on Luria-Bertani (LB) medium. The plasmids used in this study are described in Table 2. All chemicals used in media preparation and for the experiment were purchased by Merck KGaA (Darmstadt, Germany), Sigma-Aldrich Corporation (St. Louis, USA), or Hi-media (Mumbai, India). Restriction enzymes, Q5-high fidelity DNA polymerase, and Taq DNA polymerase were purchased from New England Biolabs (NEB) (Ipswich, USA) while RNases, Reverse Transcriptase, Terminal deoxynucleotidyl transferase, and T4-DNA ligase were purchased from Thermo Fisher Scientific (Waltham, USA). As per requirements, ampicillin (100 µg/mL), kanamycin (50 µg/mL), tetracycline (10 µg/mL), chloramphenicol (20 µg/mL), spectinomycin (100 µg/mL), and rifampicin (50 µg/mL) were added in culture media.

**TABLE 2:** Bacterial strains and plasmids

Strains or plasmids	Relevant properties	References/sources
Bacterial strains		
*A. brasilense* Sp7	Wild-type strain (ATCC 29729)	(34)
*E. coli* DH5α	*ΔlacU169, hsdR17, recA1, endA1, gyrA96, thiL, relA1*	Gibco/BRL
*E. coli* S.17–1	Sm^r^*, recA, thi, pro, hsdR,* RP4-2(Tc::Mu; Km:: Tn*7*)	(35)
*E. coli* BL-21(DE3)	*ompT, hsdS (r_B_^_^m_B_^_^) dcm^+,^ endA, galλ,* (DE3)	Novagen
*A. tumefaciens* EHA105	*pEHA105 (pTiBo542∆T-DNA) Succinamopine* Rif^r^	(36)
*fruA1::km* strain	*A. brasilense* Sp7 derivative with containing Km^r^ cassette in place of nucleotides *+*760 to +1,130 of the *fruA1* (AMK58_25875) gene.	This work
*fruA2::km* strain	*A. brasilense* Sp7 derivative with containing Km^r^ cassette in place of nucleotides *+*296 to +952 of the *fruA2* (AMK58___27000) gene.	This work
*fruR::km* strain	*A. brasilense* Sp7 derivative with containing Km^r^ cassette in place of nucleotides *+* 403 to +575 of the *fruR* (AMK58_25890) gene	This work
*tssF-tssG::km* strain	*A. brasilense*Sp7 derivative with containing Km^r^ cassette in place of nucleotides *+*508 to +1,893 of the *tssF* gene overlapping with *tssG* (AMK58_18850 and AMK58_18855)	This work
Plasmids		
pSUP202	ColE1 replicon, mobilizable plasmid, suicide vector suitable for *A. brasilense*; Amp^r^,Tc^r^, Cm^r^	(35)
pUC4K	Vector containing Km^r^ cassette	GE Healthcare
pMMB206	Cm^r^, broad host range, low copy no. expression vector	(37)
pCZ750	pFAJ1700 containing the KpnI-AscI *lacZ* gene from pCZ367 plasmid; Tc^r^, Amp^r^	(38)
pAK002	pBBR1MCS-3 derivative containing constitutive kanamycin resistance gene (apt) promoter	(39)
pSUP:*fruA1*	*fruA1* (AMK58_25875) gene disrupted by Km^r^ cassette cloned into EcoRI-Pstl site inpSUP202	This work
pSUP:*fruA2*	*fruA2* (AMK58_27000) gene disrupted by Km^r^ cassette cloned into EcoRI-Pstl site in pSUP202	This work
pSUP:*fruR*	*fruR* (AMK58_25890) gene disrupted by Km^r^ cassette cloned into EcoRI-Pstl site in pSUP202	This work
pSUP:*tssFG*	*fruR* (AMK58_25890) gene disrupted by Km^r^ cassette cloned into EcoRI-Pstl site in pSUP202	This work
pAK002:*fruA1*	*fruA1* (AMK58_25875) gene from *A. brasilense* Sp7 cloned into XhoI restriction site of pAK002	This work
pAK002:*fruA2*	*fruA2* (AMK58_27000) gene from *A. brasilense* Sp7 cloned into XhoI restriction site of pAK002	This work
pAK002:*lacZ*	*lacZ* (ECD_00298) gene from *E. coli* BL21 (DE3) cloned into XhoI/XmaI restriction site of pAK002	This work
*fruB1*:*lacZ*	pCZ750 derivative; Tc^r^, Amp^r^, *fruB1:lacZ*	This work
*ΔfruB1::lacZ del1*	pCZ750 derivative; Tc^r^, Amp^r^, *fruB1:lacZ* withdeletion of 50 bp (−221 to −171)	This work
*ΔfruB1::lacZ del2*	pCZ750 derivative; Tc^r^, Amp^r^, *fruB1:lacZ* with deletion of 94 bp (−221 to −127)	This work
*ΔfruB1::lacZ del3*	pCZ750 derivative; Tc^r^, Amp^r^, *fruB1:lacZ* with deletion of 123 bp (−221 to −98)	This work
*fruB2*:*lacZ*	pCZ750 derivative; Tc^r^, Amp^r^, *fruB2:lacZ*	This work
*ATPase*:*lacZ*	pCZ750 derivative; Tc^r^, Amp^r^*, ATPase:lacZ*	This work

### Bacterial growth

To compare the growth of *A. brasilense* Sp7 and its mutants in minimal malate (40 mM) medium (MMM) or minimal fructose (40 mM) medium (MFM) (19, 22, 40), ~1 mL of overnight grown culture of *A. brasilense* or its mutants were pelleted by centrifugation (in LB medium), washed with 0.85% saline, and suspended in MMM or MFM to maintain the initial OD_600_ of 0.05 (2.5 × 10^6^ cells/mL). The cultures were grown at 30°C with shaking at 180  rpm for 36 h. Growth was monitored at 4 h intervals, and growth curves were plotted. Similarly, the growth curves of complemented strains *fruA1::km* (pAK002:*fruA1*), *fruA1::km* (pAK002:*fruA2*), *fruA2::km* (pAK002:*fruA1*), *fruA2::km* (pAK002:*fruA2*), and *fruR::km* (pMMB:*fruR*) were monitored and plotted as above. The bar graphs and growth curves were prepared using the GraphPad Prism software.

### Bioinformatics analysis

The amino acid sequences of *fruA*, *fruB*, and *fruR* of *A. brasilense* Sp7 were retrieved from the KEGG database (https://www.genome.jp/kegg/). Levels of sequence identity and similarity were calculated by using BLAST (https://blast.ncbi.nlm.nih.gov/Blast.cgi), and alignment of sequences was performed by the ClustalW program of BioEdit software. Secondary structure in the promoter upstream region was analyzed using mfold software at UNAFold web server (http://www.unafold.org/mfold.php).

### Insertional inactivation of *fruA1, fruA2*, *fruR*, and *tssF-tssG* in *A. brasilense* Sp7

For insertional inactivation of *fruA1*, *fruA2*, *fruR*, and *tssF-tssG*, we used modification of an earlier described method (41). Upstream and downstream regions of each gene (∼500  bp) were PCR-amplified by using specific pairs of primers (FRUA1AF/FRUA1AR, FRUA1BF/FRUA1BR, FRUA2AF/FRUA2AR, FRUA2BF/FRUA2BR, FRURAF/FRURAR, FRURBF/FRURBR, TSSFGAF/TSSFGAR, and TSSFGBF/TSSFGBR). Sequences of the primers used for PCR amplification of different genes are listed in Table 3. Each gene was amplified in two parts, amplicons A and B which contain EcoRI/Bglll and Pstl/Bglll restriction sites, respectively. The two amplicons were first cloned separately into the pGEM-T easy vector via TA cloning, and then excised via respective restriction enzymes. Both the digested amplicons A and B were cloned in pSUP202 vector resulting in pSUP202AB, which was transformed into *E. coli* DH5α and the transformants harboring recombinant plasmids were selected on LB plates supplemented with tetracycline. Further, the recombinant plasmids were linearized with BglII, and a kanamycin resistance (*Km^r^*) gene cassette, obtained from pUC4K after digestion with BamHI, was ligated in between amplicon A and amplicon B in the BglII-linearized pSUP202AB. The resulting plasmids (pSUP:*fruA1*, pSUP:*fruA2*, pSUP:*fruR*, and pSUP:*tssFG*) were transformed into *E. coli* S17-1, and the recombinants selected on LB agar plates supplemented with kanamycin and tetracycline. The final disruption constructs were mobilized into *A. brasilense* Sp7 by conjugation using *E. coli* S17-1 as a donor. The exconjugants were selected on kanamycin-supplemented MMM plates and insertion of *Km^r^* cassette at desired locus was confirmed by PCR amplification using a gene-specific primer pair (FRUA1OF/FRUA1OF, FRUA2OF/FRUA2OF, FRUROF/FRUROF, and TSSFGOF/TSSFGOR).

**TABLE 3:** Primers used in this study

Primers	Sequence 5−3′ direction
FRUA1AF	CGGAATTCCCTGCCCTTCTGCATCAAGCC
FRUA1AR	GAAGATCTGAAGGGCAGCATGAAGGACACG
FRUA1BF	GAAGATCTCCTCGCCATGATCTACGTGG
FRUA1BR	AACTGCAGGTAGGCGAAGGCGGAGAATTTC
FRUA2AF	AACTGCAGTGAACATCAAGCTGGTGGACC
FRUA2AR	GAAGATCTCCAAGGCGTTGCCGATCACC
FRUA2BF	GAAGATCTGCTACATCGCCTATTCCATCG
FRUA2BR	CGGAATTCCGATCATCTCACGATAGACGG
FRURAF	CGGAATTCCCACCCAGTCGAACCGCTCC
FRURAR	GAAGATCTGGTCGGCGCGAAGATCAGGC
FRURBF	GAAGATCTCGGCAACACCAGCACCACGG
FRURBR	AACTGCAGGTCTACGCCTTCCTGATGCCG
TSSFGAF	AACTGCAGCGACTTCGAACCGGACCAACC
TSSFGAR	GAAGATCTCAGCCGCAGCCGCAGCACC
TSSFGBF	GAAGATCTTGCTGGAACGCGCCGCGCC
TSSFGBR	GGAATTCCGACGTCGTAATGGGTCTGCG
FRUA1OF	CCGCTCGAGACCCGCCATGGCCCCCGAC
FRUA1OR	CCGCTCGAGCTGTTACGCCGTCACCACGC
FRUA2OF	CCGCTCGAGGATGCTTCAGGTCTCCGAATCG
FRUA2OR	CCGCTCGAGGCTGTGGTGATTCCAGGTCG
FRUROF	CGGGATCCATGAGCGACAGCGCCAAAGC
FRUROR	AACTGCAGCGCAACCACTCACCCCACC
LACZB21F	CCGCTCGAGCATGACCATGATTACGGATTCACTGG
LACZB21R	TCCCCCCGGGTTATTTTTGACACCAGACCAACTG
ZFRUB1F	TGCTCTAGACCATGGCGGGTCCGTCTCC
ZFRUB1R	CCCAAGCTTCTCATGATGATCCGGTCACCG
ZFRUB1.1R	CCCAAGCTTTCCCTTAGGGCCAATCCACCG
ZFRUB1.2R	CCCAAGCTTAAGGAGTCCGCGGATTCAATGC
ZFRUB1.3R	CCCAAGCTTGTTGACAGAACCAGATGTAATCG
ZFRUB2F	TGCTCTAGACGCTCGTCCTCCCCCAGC
ZFRUB2R	CCCAAGCTTCCCTGCGCGAATCTGTCCCG
ZATPASF	TGCTCTAGATGATCCTCGCGGCAGCGTTCA
ZATPASR	CCCAAGCTTCCCGCCTTCCGTCCGCCG
GSP1	CGTCGGCCACCAGAT
GSP2	GGCCGGTGGGGTAATC
GSP3	CCAGTCGAACCGCTCCGTCA
Oligo-dT anchor primer	GACCACGCGTATCGATGTCGACTTTTTTTTTTTTTTTTTTTTTTTV
Anchor primer	GACCACGCGTATCGATGTCGAC

### Identification of fructose-induced proteins

For identifying proteins that were expressed in *A. brasilense* Sp7 when it is grown with fructose or malate as sole source of carbon, we used a method described earlier (42). For this, the culture of *A. brasilense* Sp7 was grown in MMM and MFM, and after the cultures attained 1.0 OD_600_ (5 × 10^8^ cells/mL), cells were pelleted by centrifugation. Total proteins of the samples were prepared by sonication and centrifugation. Further, each sample of the proteins was treated with 5 mM Tris(2-carboxyethyl) phosphine hydrochloride, alkylated with 50 mM iodoacetamide and then 50 µg of protein lysate was digested with 500 ng of the trypsin, for 14 h at 37°C. The digests were cleaned using a C18 silica cartridge to remove salts and other impurities followed by drying in a SpeedVac. The dried pellet was then resuspended in buffer A (5% acetonitrile and 0.1% formic acid). All the experiments were performed using the EASY-nLC 1000 system (Thermo Fisher Scientific) coupled to OrbitrapQ-Exactive (Thermo) equipped with a nano-electrospray ion source. Peptide mixtures were resolved using a 60 cm Viper column filled with C18 resin. The peptides were loaded with buffer A and eluted with a 0–40% gradient of buffer B (95% acetonitrile and 0.1% formic acid) at a flow rate of 250 mL/min for 100 min. Raw files generated after processing the samples were analyzed using Proteome Discoverer (v2.2) against the UniProt *A. brasilense* Sp7 reference proteome database. Carbamidomethyl on cysteine was considered a fixed modification and oxidation of methionine and N-terminal acetylation were considered variable modifications for database searches. Both the peptide spectrum match and protein false discovery rate were set to 0.01.

### Recombinant plasmid construction

For complementation studies and for creating *fruA1* and *fruA2* overexpressing strains, genes encoding *fruA*1 and *fruA2* were PCR-amplified using primer-pairs (FRUA1OF/FRUA1OR and FRUA2OF/FRUA2OR) containing XhoI restriction sites in their 5′ and 3′ ends. Further, amplicons were cloned in a modified pBBR3MCS1 vector (pAK002) (39), under the constitutive control of *Km* promoter. to construct the recombinant plasmids pAK002:*fruA1* and pAK002:*fruA2*. Similarly, promoterless β-galactosidase gene of *E. coli* BL21 was PCR amplified by using the primer set LACZB21F/LACZB21R and cloned into pAK002 vector to construct the recombinant plasmid pAK002:*lacZ*. The *fruR* gene was amplified by PCR using the primer set FRUROF/FRUROR and cloned into an IPTG inducible, low-copy number vector pMMB206 to construct the recombinant plasmid pMMB:*fruR*.

To study the regulation of expression of the two Fru-PTS in *A. brasilense*, the upstream region of the three genes *fruB1*, *fruB2*, and *ATPase* were PCR-amplified with the help of three primer pairs (ZFRUB1F/ZFRUB1R, ZFRUB2F/ZFRUB2R, and ZATPASF/ZATPASR) containing XbaI and HindIII restriction sites in their forward and reverse primers, respectively. Further, amplicons were cloned into pCZ750 vector at the same restriction sites present upstream of the *lacZ* reporter resulting in three recombinant plasmids (pCZ:*fruB*1, pCZ:*fruB*2, and pCZ:*ATPase*). Similarly, deletion derivatives of the *fruB1* promoter were also prepared by using three sets of primers (ZFRUB1F/ZFRUB1.1R, ZFRUB1F/ZFRUB1.2R, and ZFRUB1F/ZFRUB1.3R), cloned in XbaI and HindIII restriction sites of pCZ750 vector to construct the three recombinant plasmids (*ΔfruB1::lacZ del1*, *ΔfruB1::lacZ del2*, and *ΔfruB1::lacZ del3*).

### β-Galactosidase assay

The WT and recombinant strains of *A. brasilense* Sp7 harboring (pCZ:*fruB*1, pCZ:*fruB*2, pCZ:*ATPase*, *ΔfruB1::lacZ del1*, *ΔfruB1::lacZ del2*, and *ΔfruB1::lacZ del3*) plasmids were grown overnight in LB medium with shaking at 180 rpm and 30°C. Aliquots (~1 mL) of overnight cultures were harvested, washed, and resuspended in MMM and MFM. Cultures were grown for 8 h at 30°C and 180 rpm. After 8 h, cells were pelleted by centrifugation and resuspended in a lysis buffer (50 mM phosphate buffer [pH 7.0], 0.1% SDS, 0.27% β-mercaptoethanol, and 100 µL chloroform) for the assay of β-galactosidase activity, which was measured as described earlier (43) by using the formula: 1,000 × (OD_420_  × 1.75 − OD_550_)/time of reaction (in min) × volume of culture assayed.

### Mapping of the TSS

The TSS or 5′ end of *fruB1* mRNA was identified by 5′ RACE as previously described (42). Briefly, *A. brasilense* Sp7 was grown to log phase in minimal fructose medium, and RNA was isolated using the TRIzol method. After DNase treatment, 5 µg of total RNA was reverse-transcribed into cDNA by reverse transcriptase using gene-specific reverse primer 1 (GSP1), followed by cDNA purification and poly(dA)-tailing by terminal deoxynucleotide transferase. The poly(dA)-tailed cDNA was then PCR amplified by high-fidelity DNA polymerase using GSP2 and oligo-dT anchor primers. The amplicons obtained were used as templates in the next round of nested PCR using anchor and GSP3 primers. The final PCR product was then cloned into the pGEM-T Easy vector (Promega), and the nucleotide sequence was determined by the chain termination method.

### Estimation of d-fructose utilization during growth

d-fructose consumption during bacterial growth was monitored by using a colorimetric method (44) with some modification. Overnight cultures of *A. brasilense* Sp7, *fruA1::km*, and *fruA2::km* mutants and their derivatives grown in LB broth were pelleted, washed with MFM and reinoculated in MFM with an initial OD_600_ of 0.05 (2.5 × 10^6^ cells/mL). Bacterial growth was monitored for up to 36 h, after which the culture was centrifuged to pellet the cells and cell-free supernatants (in triplicates) of spent medium were used for the estimation of residual d-fructose. For this, 200 µL spent medium was mixed with 5 µL phenol (80%) and 500 µL concentrated sulfuric acid in microtubes (45). Samples were kept in a water bath at 30°C for 20 min, after which the color development was recorded at OD_490_ using a spectrophotometer. For estimation of unknown concentrations of d-fructose, a standard curve was plotted using 1, 2, 4, 16, and 32 µg of d-fructose, and a slope of the curve was derived.

### Chemotaxis assays

For chemotactic assay, *A. brasilense* Sp7 and *fruA1::km*, *fruA2::km*, and *fruR::km* mutants were grown overnight in MMM. An equal number of cells, 6.25 × 10^9^ cells (12.5 OD_600_ cells), were pelleted by centrifugation, washed two times, and re-suspended in 25 mL of minimal medium with 0.3% agar plus triphenyl tetrazolium chloride (TTC: 66 µg/mL) lacking any carbon source. Plates were dried for 10 min at room temperature under a laminar airflow hood. Then, a drop of 2 µL of 1 M fructose or 1 M malate solution was spotted on a 10 mm sterile filter disc in the center of the plates. The formation of a chemotactic ring around the disc was recorded after 3 h of incubation.

### Contact-dependent killing of *E. coli* and *A. tumefaciens* by *A. brasilense* Sp7

For demonstration of the fructose-specific and contact-dependent killing of *E. coli* S17-1 and *A. tumefaciens* EHA105 by *A. brasilense* Sp7, we first cloned a complete β-galactosidease gene (*lacZ*) from *E. coli* BL21 in a broad host range vector (pAK002) under constitutively expressed promoter. The resultant recombinant plasmid was designated as pAK002:*lacZ*, which was mobilized in to *E. coli* S17-1 and *A. tumefaciens* EHA105, via transformation and electroporation, respectively. We then prepared plates of LB agar, minimal fructose agar (MFA) and minimal glycerol agar (MGA) containing 1% agar and X-gal (40 µg/mL). Further, 1 OD_600_ cells (5 × 10^8^ cells) of overnight grown culture (in LB medium) of *A. brasilense* Sp7 were pelleted, washed, and mixed in 5 mL of LBA, MFA, and MGM (containing only 0.5% agar) and poured on media plates to prepare the lawn of *A. brasilense* Sp7. Plates were dried under a laminar airflow hood for 10 min and then incubated further at 30°C. After 4 h, from the pre-grown log phase cultures of recombinant *E. coli* S17-1 (pAK002:*lacZ*) and *A. tumefaciens* EHA105 (pAK002:*lacZ*), 1 OD_600_ cells of each were pelleted, washed, and used for the preparation of 5 µL drops of several dilutions. Drops of different dilutions of *E. coli* and *A. tumefaciens* EHA105 were placed on the pre-made lawn of *A. brasilense* Sp7. Growth of the colonies of *E. coli* S17-1 (pAK002:*lacZ*) and *A. tumefaciens* EHA105 (pAK002:*lacZ*) on the plates was monitored and the final images taken after 4 days. To ensure the optimal growth of *E. coli* S17-1, agar medium was supplemented with thiamine and proline. To examine the ability of *tssFG::km* mutant for contact-dependent killing of *E. coli* and *A. tumefaciens*, we used *tssFG::km* mutant of *A. brasilense* Sp7 to prepare its lawn for the contact-dependent killing experiment.

### Statistical analysis

Experiments on the measurement of growth and d-fructose consumption were performed in triplicates in three independent experiments. The mean significant values were determined by using SPSS 17 package software. Analysis of the variance followed by Duncan’s multiple comparison tests (46) was used to analyze the significance between more than two treatments. Differences were considered significant at *P* < 0.05.

## Data Availability

The proteomics data have been deposited in the EMBL PRIDE database with accession number PXD055876.

## SUPPLEMENTAL MATERIAL

Supplemental figures aem.00828-24-s0001.pdf Figures S1 and S2.

Table S1 aem.00828-24-s0002.xlsx List of expressed proteins of A. brasilense Sp7 grown with malate or fructose as the sole carbon source.

ASM does not own the copyrights to Supplemental Material that may be linked to, or accessed through, an article. The authors have granted ASM a non-exclusive, world-wide license to publish the Supplemental Material files. Please contact the corresponding author directly for reuse.

## References

[1] Postma PW, Lengeler JW, Jacobson GR. 1993. Phosphoenolpyruvate:carbohydrate phosphotransferase systems of bacteria. Microbiol Rev 57:543–594. doi:10.1128/mr.57.3.543-594.1993 8246840 PMC372926

[2] Yoon CK, Kang D, Kim MK, Seok YJ. 2021. Vibrio cholerae FruR facilitates binding of RNA polymerase to the fru promoter in the presence of fructose 1-phosphate. Nucleic Acids Res 49:1397–1410. doi:10.1093/nar/gkab013 33476373 PMC7897506

[3] Deutscher J, Francke C, Postma PW. 2006. How phosphotransferase system-related protein phosphorylation regulates carbohydrate metabolism in bacteria. Microbiol Mol Biol Rev 70:939–1031. doi:10.1128/MMBR.00024-06 17158705 PMC1698508

[4] Tchieu JH, Norris V, Edwards JS, Saier MH Jr. 2001. The complete phosphotransferase system in Escherichia coli. J Mol Microbiol Biotechnol 3:329–346. 11361063

[5] Granot D, David-Schwartz R, Kelly G. 2013. Hexose kinases and their role in sugar-sensing and plant development. Front Plant Sci 4:44. doi:10.3389/fpls.2013.00044 23487525 PMC3594732

[6] Saier MH Jr, Ramseier TM. 1996. The catabolite repressor/activator (Cra) protein of enteric bacteria. J Bacteriol 178:3411–3417. doi:10.1128/jb.178.12.3411-3417.1996 8655535 PMC178107

[7] Kornberg HL. 2001. Routes for fructose utilization by Escherichia coli. J Mol Microbiol Biotechnol 3:355–359. https://www.caister.com/backlist/jmmb/v/v3/v3n3/04.pdf. 11361065

[8] Pedraza RO. 2015. Siderophores production by *Azospirillum*: biological importance, assessing methods and biocontrol activity, p 251–262. In Handbook for Azospirillum: technical issues and protocols. Springer International Publishing, Cham.

[9] Delaporte-Quintana P, Lovaisa NC, Rapisarda VA, Pedraza RO. 2020. The plant growth promoting bacteria Gluconacetobacter diazotrophicus and Azospirillum brasilense contribute to the iron nutrition of strawberry plants through siderophores production. Plant Growth Regul 91:185–199. doi:10.1007/s10725-020-00598-0

[10] Cassán F, Coniglio A, López G, Molina R, Nievas S, de Carlan CLN, Donadio F, Torres D, Rosas S, Pedrosa FO, de Souza E, Zorita MD, de-Bashan L, Mora V. 2020. Everything you must know about Azospirillum and its impact on agriculture and beyond. Biol Fertil Soils 56:461–479. doi:10.1007/s00374-020-01463-y

[11] Tien TM, Gaskins MH, Hubbell DH. 1979. Plant growth substances produced by Azospirillum brasilense and their effect on the growth of pearl millet (Pennisetum americanum L.). Appl Environ Microbiol 37:1016–1024. doi:10.1128/aem.37.5.1016-1024.1979 16345372 PMC243341

[12] Venkateswarlu B, Rao AV. 1983. Response of pearl millet to inoculation with different strains of Azospirillum brasilense. Pl Soil 74:379–386. doi:10.1007/BF02181355

[13] Tarrand JJ, Krieg NR, Döbereiner J. 1978. A taxonomic study of the Spirillum lipoferum group, with descriptions of a new genus, Azospirillum gen. nov. and two species, Azospirillum lipoferum (Beijerinck) comb. nov. and Azospirillum brasilense sp. nov. Can J Microbiol 24:967–980. doi:10.1139/m78-160 356945

[14] Das A, Mishra AK. 1983. Utilization of fructose by Azospirillum brasilense. Can J Microbiol 29:1213–1217. doi:10.1139/m83-185

[15] Gupta KD, Ghosh S. 1984. Identification of a phosphoenolpyruvate: fructose 1-phosphotransferase system in Azospirillum brasilense. J Bacteriol 160:1204–1206. doi:10.1128/jb.160.3.1204-1206.1984 6501230 PMC215847

[16] Goebel EM, Krieg NR. 1984. Fructose catabolism in Azospirillum brasilense and Azospirillum lipoferum. J Bacteriol 159:86–92. doi:10.1128/jb.159.1.86-92.1984 6735986 PMC215596

[17] Martinez-Drets G, Del Gallo M, Burpee C, Burris RH. 1984. Catabolism of carbohydrates and organic acids and expression of nitrogenase by azospirilla. J Bacteriol 159:80–85. doi:10.1128/jb.159.1.80-85.1984 6588050 PMC215595

[18] Mukherjee A, Ghosh S. 1987. Regulation of fructose uptake and catabolism by succinate in Azospirillum brasilense. J Bacteriol 169:4361–4367. doi:10.1128/jb.169.9.4361-4367.1987 2957360 PMC213753

[19] Sadasivan LA, Neyra CA. 1985. Flocculation in Azospirillum brasilense and Azospirillum lipoferum: exopolysaccharides and cyst formation. J Bacteriol 163:716–723. doi:10.1128/jb.163.2.716-723.1985 3894333 PMC219180

[20] Sadasivan L, Neyra CA. 1987. Cyst production and brown pigment formation in aging cultures of Azospirillum brasilense ATCC 29145. J Bacteriol 169:1670–1677. doi:10.1128/jb.169.4.1670-1677.1987 3104311 PMC211998

[21] Burdman S, Jurkevitch E, Schwartsburd B, Hampel M, Okon Y. 1998. Aggregation in Azospirillum brasilense: effects of chemical and physical factors and involvement of extracellular components. Microbiol (Reading) 144 ( Pt 7):1989–1999. doi:10.1099/00221287-144-7-1989 9695932

[22] Singh VS, Dubey BK, Pandey P, Rai S, Tripathi AK. 2021. Cometabolism of ethanol in Azospirillum brasilense Sp7 is mediated by fructose and glycerol and regulated negatively by an alternative sigma factor RpoH2. J Bacteriol 203:e0026921. doi:10.1128/JB.00269-21 34570625 PMC8604076

[23] Brunet YR, Zoued A, Boyer F, Douzi B, Cascales E. 2015. The type VI secretion TssEFGK-VgrG phage-like baseplate is recruited to the TssJLM membrane complex via multiple contacts and serves as assembly platform for tail tube/sheath polymerization. PLoS Genet 11:e1005545. doi:10.1371/journal.pgen.1005545 26460929 PMC4604203

[24] Cassan FD, Coniglio A, Amavizca E, Maroniche G, Cascales E, Bashan Y, de-Bashan LE. 2021. The Azospirillum brasilense type VI secretion system promotes cell aggregation, biocontrol protection against phytopathogens and attachment to the microalgae Chlorella sorokiniana. Environ Microbiol 23:6257–6274. doi:10.1111/1462-2920.15749 34472164

[25] Mukherjee T, Kumar D, Burriss N, Xie Z, Alexandre G. 2016. Azospirillum brasilense chemotaxis depends on two signaling pathways regulating distinct motility parameters. J Bacteriol 198:1764–1772. doi:10.1128/JB.00020-16 27068592 PMC4886762

[26] Huang Z, Pan X, Xu N, Guo M. 2019. Bacterial chemotaxis coupling protein: structure, function and diversity. Microbiol Res 219:40–48. doi:10.1016/j.micres.2018.11.001 30642465

[27] Ganusova EE, Vo LT, Abraham PE, O’Neal Yoder L, Hettich RL, Alexandre G. 2021. The Azospirillum brasilense core chemotaxis proteins CheA1 and CheA4 link chemotaxis signaling with nitrogen metabolism. mSystems 6:10–128. doi:10.1128/mSystems.01354-20 PMC8561660 33594007

[28] Chavarría M, Fuhrer T, Sauer U, Pflüger-Grau K, de Lorenzo V. 2013. Cra regulates the cross-talk between the two branches of the phosphoenolpyruvate: phosphotransferase system of Pseudomonas putida. Environ Microbiol 15:121–132. doi:10.1111/j.1462-2920.2012.02808.x 22708906

[29] Browning DF, Busby SJ. 2004. The regulation of bacterial transcription initiation. Nat Rev Microbiol 2:57–65. doi:10.1038/nrmicro787 15035009

[30] Bingle LE, Bailey CM, Pallen MJ. 2008. Type VI secretion: a beginner’s guide. Curr Opin Microbiol 11:3–8. doi:10.1016/j.mib.2008.01.006 18289922

[31] Cascales E. 2008. The type VI secretion toolkit. EMBO Rep 9:735–741. doi:10.1038/embor.2008.131 18617888 PMC2515208

[32] Carobbi A, Di Nepi S, Fridman CM, Dar Y, Ben-Yaakov R, Barash I, Salomon D, Sessa G. 2022. An antibacterial T6SS in Pantoea agglomerans pv. betae delivers a lysozyme-like effector to antagonize competitors. Environ Microbiol 24:4787–4802. doi:10.1111/1462-2920.16100 35706135 PMC9796082

[33] Vanstockem M, Michiels K, Vanderleyden J, Van Gool AP. 1987. Transposon mutagenesis of Azospirillum brasilense and Azospirillum lipoferum: physical analysis of Tn5 and Tn5-Mob insertion mutants. Appl Environ Microbiol 53:410–415. doi:10.1128/aem.53.2.410-415.1987 16347289 PMC203674

[34] Nur I, Steinitz YL, Okon Y, Henis Y. 1981. Carotenoid composition and function in nitrogen-fixing bacteria of the genus Azospirillum. Microbiol (Reading, Engl) 122:27–32. doi:10.1099/00221287-122-1-27

[35] Simon RU, Priefer U, Pühler A. 1983. A broad host range mobilization system for in vivo genetic engineering: transposon mutagenesis in gram negative bacteria. Nat Biotechnol 1:784–791. doi:10.1038/nbt1183-784

[36] Hood EE, Gelvin SB, Melchers LS, Hoekema A. 1993. New Agrobacterium helper plasmids for gene transfer to plants. Transgenic Res 2:208–218. doi:10.1007/BF01977351

[37] Morales VM, Bäckman A, Bagdasarian M. 1991. A series of wide-host-range low-copy-number vectors that allow direct screening for recombinants. Gene 97:39–47. doi:10.1016/0378-1119(91)90007-x 1847347

[38] Dombrecht B, Vanderleyden J, Michiels J. 2001. Stable RK2-derived cloning vectors for the analysis of gene expression and gene function in Gram-negative bacteria. Mol Plant Microbe Interact 14:426–430. doi:10.1094/MPMI.2001.14.3.426 11277442

[39] Pandey P, Dubey AP, Mishra S, Singh VS, Singh C, Tripathi AK. 2022. β-Lactam resistance in Azospirillum baldaniorum Sp245 is mediated by lytic transglycosylase and β-Lactamase and regulated by a cascade of RpoE7→RpoH3 sigma factors. J Bacteriol 204:e0001022. doi:10.1128/jb.00010-22 35352964 PMC9017315

[40] Okon Y, Albrecht SL, Burris RH. 1977. Methods for growing Spirillum lipoferum and for counting it in pure culture and in association with plants. Appl Environ Microbiol 33:85–88. doi:10.1128/aem.33.1.85-88.1977 16345192 PMC170579

[41] Singh VS, Dubey AP, Gupta A, Singh S, Singh BN, Tripathi AK. 2017. Regulation of a glycerol-induced quinoprotein alcohol dehydrogenase by σ^54^ and a LuxR-type regulator in Azospirillum brasilense Sp7. J Bacteriol 199:10–128. doi:10.1128/JB.00035-17 PMC5472811 28439037

[42] Dubey AP, Pandey P, Singh VS, Mishra MN, Singh S, Mishra R, Tripathi AK. 2020. An ECF41 family σ factor controls motility and biogenesis of lateral flagella in Azospirillum brasilense Sp245. J Bacteriol 202:10–128. doi:10.1128/JB.00231-20 PMC8404707 32513682

[43] Miller JH. 1972. Experiments in molecular genetics. Cold Spring Harbor Laboratory Press, Cold Spring Harbor, NY.

[44] DuBois M, Gilles KA, Hamilton JK, Rebers PA, Smith F. 1956. Colorimetric method for determination of sugars and related substances. Anal Chem 28:350–356. doi:10.1021/ac60111a017

[45] Singh VS, Dubey BK, Rai S, Singh SP, Tripathi AK. 2022. Engineering D-glucose utilization in Azospirillum brasilense Sp7 promotes rice root colonization. Appl Microbiol Biotechnol 106:7891–7903. doi:10.1007/s00253-022-12250-0 36334127

[46] Duncan DB. 1955. Multiple range and multiple F tests. Biometrics 11:1. doi:10.2307/3001478

