# Supplementary material for: Identification and functional characterization of a fructose-inducible phosphotransferase system in Azospirillum brasilense Sp7
Source: Appl Environ Microbiol. 2025 Jan 16;91(2):e00828-24. doi: 10.1128/aem.00828-24 (PMC11837500; doi:10.1128/aem.00828-24)
Supplement: Supplemental figures — Figures S1 and S2. [file aem.00828-24-s0001.pdf]

<u>Supplementary Material</u>

Supplemental Table S1: List of expressed proteins of *A. brasilense* Sp7 grown with malate (F1: control, F2: control and F3: control) or fructose (F4: Sample, F5: Sample and F6: Sample) as the sole carbon source analyzed by using Thermo Proteome Discoverer. **Sheet 1** contains all the expressed proteins obtained by proteome analysis. **Sheet 2** lists 576 proteins that were upregulated by >2 fold. **Sheet 3** lists 139 proteins that were detected in fructose grown cultures only. Description, q-value, Molecular weight; MW [kDa], Pfam IDs, Entrez Gene ID, Gene ID, Abundance Ratio: Sample/Control (highlighted in yellow), Abundance Ratio (log2): Sample/Control and p-value are mentioned in list.

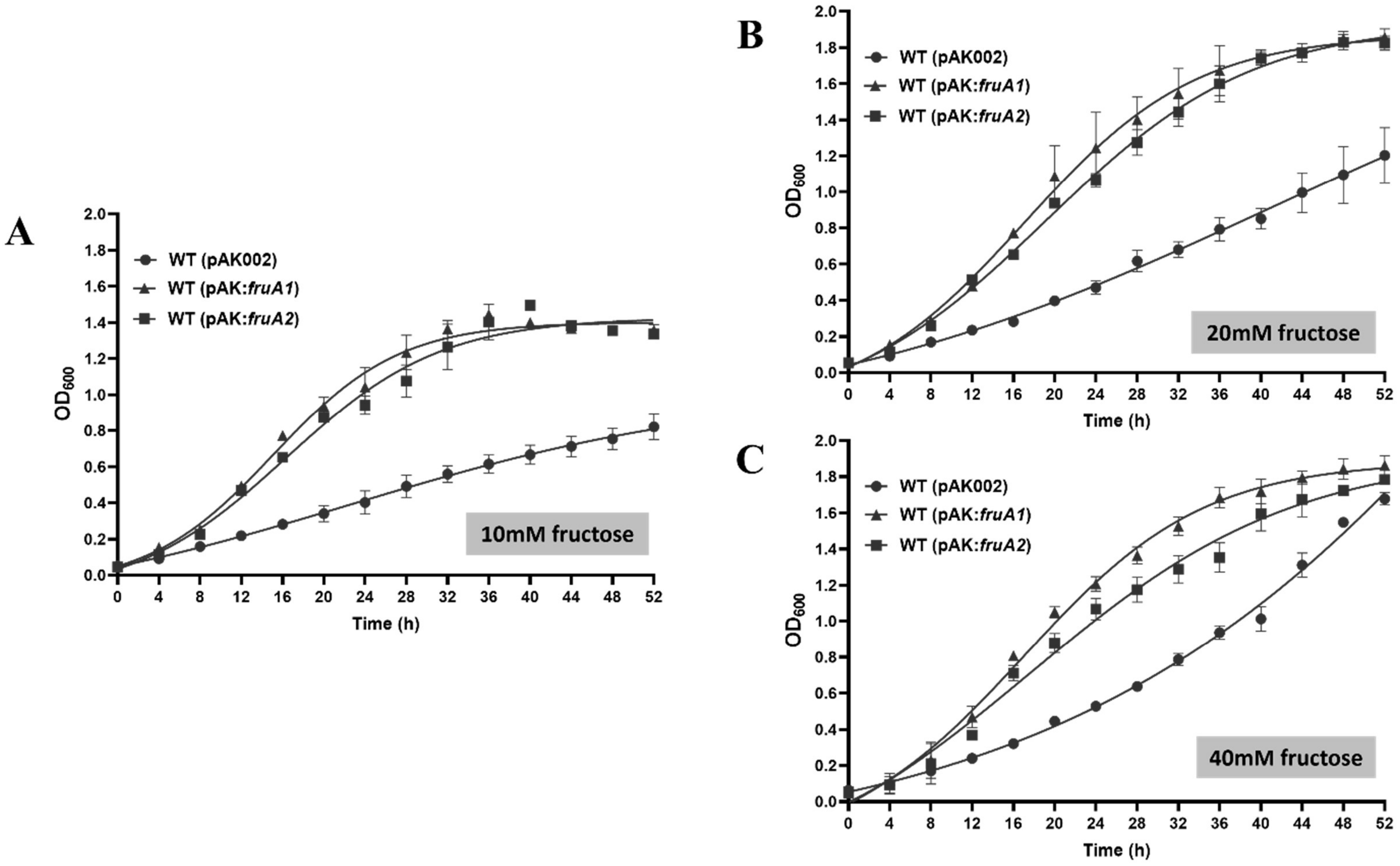

Fig. S1: Comparison of the growth of WT (pAK002), WT (pAK002:*fruA1*) and WT (pAK002:*fruA2*) in minimal fructose medium containing 10mM **(A)**, 20mM **(B)** and 40mM **(C)** fructose as the sole carbon source.

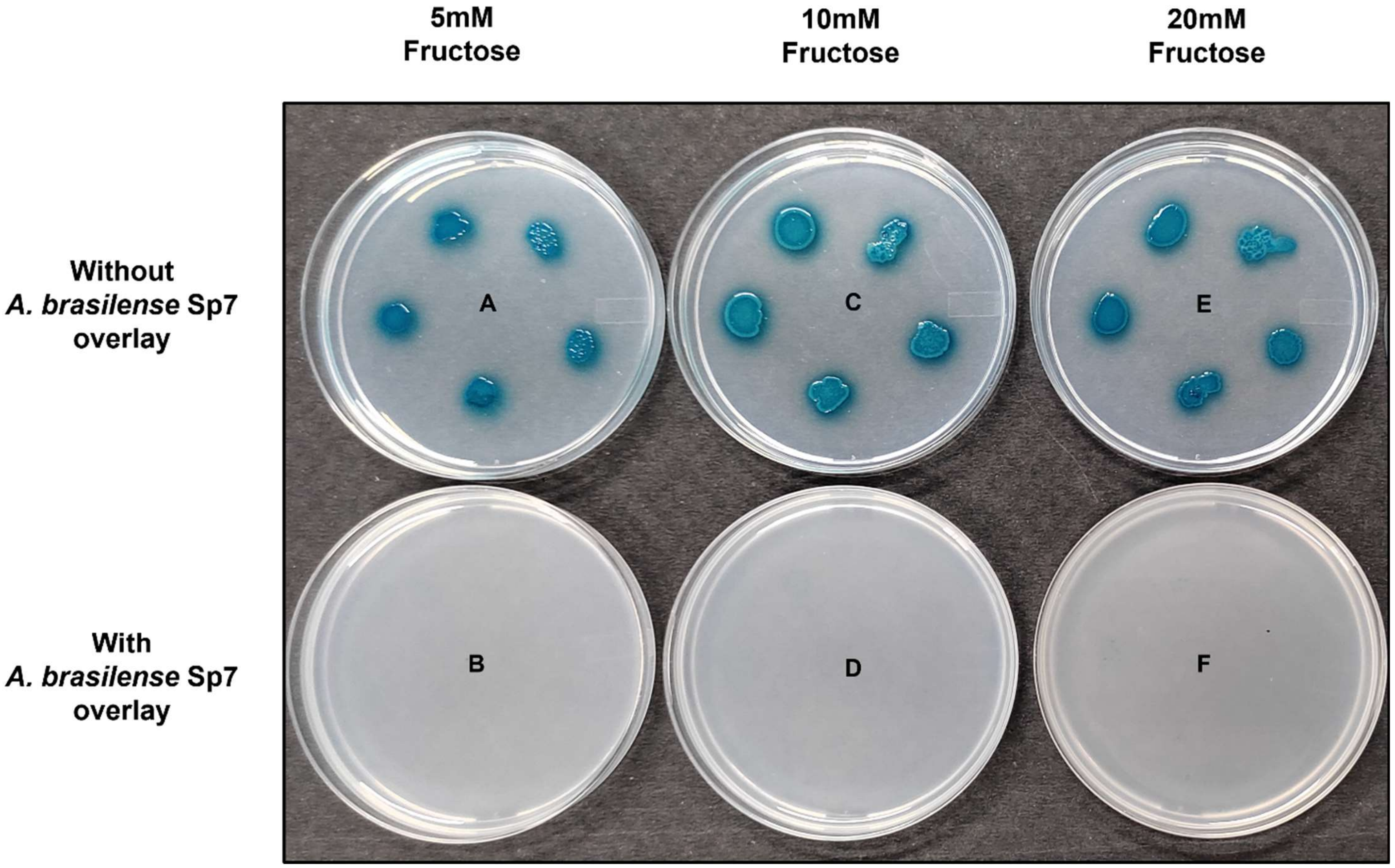

Fig. S2: Effect of different concentrations of fructose on the contact-dependent growth inhibition by *A. brasilense* Sp7 on MFM plates containing 5mM, 10mM and 20mM fructose. Plates **A**, **C** and **E** shows growth of *E. coli* S17-1 (pAK002:*lacZ*), when overlaid with soft agar lacking *A. brasilense* Sp7 on 5mM, 10mM and 20mM fructose, respectively. Plates **B**, **D** and **F** shows inhibition of growth of *E. coli* S17-1 (pAK002:*lacZ*) under same condition when overlaid with soft agar containing *A. brasilense* Sp7 on 5mM, 10mM and 20mM fructose, respectively. Plates were supplemented with thiamine and proline. Blue colonies on the plates show growth of different dilutions of the culture containing approximately 8×10^4, 4×10^4, 2×10^4, 1×10^4, 0.5×10^4 cells of *E. coli* S17-1 (pAK002:*lacZ*) spotted (anti-clockwise) on an overlay of soft agar, and plates were observed after 4 days of incubation at 30°C.
